# Protein Kinase C Isozymes Associated With Relapse Free Survival in Non-Small Cell Lung Cancer Patients

**DOI:** 10.3389/fonc.2020.590755

**Published:** 2020-11-25

**Authors:** Ann Rita Halvorsen, Mads Haugland Haugen, Åsa Kristina Öjlert, Marius Lund-Iversen, Lars Jørgensen, Steinar Solberg, Gunhild M. Mælandsmo, Odd Terje Brustugun, Åslaug Helland

**Affiliations:** ^1^ Department of Cancer Genetics, Institute for Cancer Research, Oslo University Hospital-Radiumhospitalet, Oslo, Norway; ^2^ Department of Clinical Medicine, University of Oslo, Oslo, Norway; ^3^ Department of Tumor Biology, Institute for Cancer Research, Oslo University Hospital-Radiumhospitalet, Oslo, Norway; ^4^ Department of Pathology, Oslo University Hospital-Radiumhospitalet, Oslo, Norway; ^5^ Department of Cardiothoracic Surgery, Oslo University Hospital-Rikshospitalet, Oslo, Norway; ^6^ Faculty of Health Sciences, Institute of Medical Biology, UiT-Arctic University of Norway, Tromso, Norway; ^7^ Section of Oncology, Drammen Hospital, Vestre Viken Hospital Trust, Drammen, Norway; ^8^ Department of Oncology, Oslo University Hospital-Radiumhospitalet, Oslo, Norway

**Keywords:** non-small cell lung cancer, protein profiling, expression subtypes, protein kinase C, adenocarcinoma, neuroendocrine markers, relapse free survival

## Abstract

**Introduction:**

Protein expression is deregulated in cancer, and the proteomic changes observed in lung cancer may be a consequence of mutations in essential genes. The purpose of this study was to identify protein expression associated with prognosis in lung cancers stratified by smoking status, molecular subtypes, and *EGFR-*, *TP53-*, and *KRAS*-mutations.

**Methods:**

We performed profiling of 295 cancer-relevant phosphorylated and non-phosphorylated proteins, using reverse phase protein arrays. Biopsies from 80 patients with operable lung adenocarcinomas were analyzed for protein expression and association with relapse free survival (RFS) were studied.

**Results:**

Spearman’s rank correlation analysis identified 46 proteins with significant association to RFS (p<0.05). High expression of protein kinase C (PKC)-α and the phosporylated state of PKC-α, PKC-β, and PKC-δ, showed the strongest positive correlation to RFS, especially in the wild type samples. This was confirmed in gene expression data from 172 samples. Based on protein expression, unsupervised hierarchical clustering separated the samples into four subclusters enriched with the molecular subtypes terminal respiratory unit (TRU), proximal proliferative (PP), and proximal inflammatory (PI) (p=0.0001). Subcluster 2 contained a smaller cluster (2a) enriched with samples of the subtype PP, low expression of the PKC isozymes, and associated with poor RFS (p=0.003) compared to the other samples. Low expression of the PKC isozymes in the subtype PP and a reduced relapse free survival was confirmed with The Cancer Genome Atlas (TCGA) lung adenocarcinoma (LUAD) samples.

**Conclusion:**

This study identified different proteins associated with RFS depending on molecular subtype, smoking- and mutational-status, with PKC-α, PKC-β, and PKC-δ showing the strongest correlation.

## Introduction

Proteins are the functional players driving both normal and disease processes. Some of the most important types of mutations in lung cancer occur in epidermal growth factor (*EGFR)*, tumor protein p53 (*TP53*), and Kirsten rat sarcoma viral oncogene homolog (*KRAS*). Mutations in these genes may lead to changes in many interacting pathways, leading to significantly altered protein expression. They are known to influence treatment response and regarded as essential for progression of lung cancer (1). Some of the changes in protein expression observed in lung cancer are a consequence of mutations in essential driver genes, and targeted therapy is usually efficient for these subgroups of patients (2).

Patients with somatic genomic alterations in the *EGFR* gene are routinely treated with *EGFR* inhibitors, which have improved the outcome for this patient group (3). Mutations in *KRAS* leads to constitutively and persistent stimulus-independent activation of downstream pathways affecting tumor growth, proliferation, and survival. Developing treatment targeting KRas has proved to be complicated, but ongoing studies investigate inhibition of effector-molecules downstream of KRas, including (extracellular signal-regulated kinase) ERK and mitogen-activated extracellular signal-regulated kinase (MEK) (4). Nevertheless, MEK inhibitors are associated with early development of resistance due to crosstalk with other signaling pathways which make this approach challenging (5). So far, no efficient therapy to re-establish the function of p53 is in clinical use, but studies with reactivation of p53 have been performed (6). In order to improve outcome for lung cancer patients, stratification based on alterations in essential genes and the affected pathways may lead to better treatment strategies and increased response rate.

To sub-classify non-small cell lung cancer (NSCLC), intrinsic molecular subtypes have been explored based on gene expression profiling. Three subtypes have been identified for the adenocarcinomas, namely the bronchioid, magnoid, and squamoid subtype, later re-named to terminal respiratory unit (TRU), proximal proliferative (PP), and proximal inflammatory (PI), respectively (7–9). The TRU subtype is most common among females and never-smokers, and often includes tumors with *EGFR* mutations and a less invasive phenotype. Early stages of the TRU subtype imply better prognosis. Gene expression profiles related to biological processes involved in excretion, asthma, and surfactants are associated with the TRU subtype. The PP subtype is reported with a high frequency of *KRAS* and *TP53* mutations, over-expression of DNA repair genes and is often found in heavy smokers. The PI subtype is recognized with over-expression of defense response genes such as CXCL10 and is most common in high grade tumors (7). At late stages, the PI subtype is associated with better survival compared to the other subtypes (8). Chromosomal instability (CIN), copy number alterations, and genomewide DNA methylations are also reported to differ among the three subtypes, with the PP subtype having the highest CIN (8). So far, protein expression are reported to only partially correlate with the molecular subtypes of adenocarcinoma (9). In order to treat lung cancer patients more efficiently, groups of patients who share common biological features such as mutations or pathway alterations should be identified and treated with drugs optimized for their subgroup. It has been known for a long time that never-smoking NSCLCs are recognized with a different underlying biology compared to ever-smokers. However, except from a handful of known genetic aberrations such as *EGFR* mutations and anaplastic lymphoma receptor tyrosine kinase (*ALK*) rearrangement, no subgroups of NSCLC are stratified for optimized treatment based on the molecular profile of the tumor.

The purpose of this study of lung adenocarcinoma, was to identify differences in protein expression levels associated with prognosis, stratified on *EGFR*, *TP53* and *KRAS* mutations status, and smoking status.

## Methods

Patients diagnosed with operable NSCLC adenocarcinoma from 2006 to 2011, were included and analyzed for protein expression (n=80) in the Oslo cohort. The patients underwent curatively intended surgical resection at Rikshospitalet, Oslo University Hospital, Norway. Tumor samples were snap frozen in liquid nitrogen and stored at −80°C until protein extraction. Pathological stage, mutation status and smoking status are outlined in **Table 1**. All the samples were classified as adenocarcinomas. Never-smokers are defined as those who have smoked less than 100 cigarettes. Out of the 80 patients, 10 died from non-lung cancer related causes, and were excluded from Spearman’s test. One sample did not have gene expression data, and was excluded for correlation analysis between gene expression and protein data.

**Table 1:** Age, gender, adjuvant chemotherapy, mutation status, smoking history, and stage are displayed for the patients analyzed for protein expression (n=80).

Condition		Number
Age	*Median (range)*	67.2 (44.8–84.1)
Gender	*Female (male)*	43 (37)
Adjuvant chemotherapy	*Chemo (no chemo) (nd)*	21 (47) (12)
*EGFR*	*Mutated (WT)*	9 (71)
*KRAS*	*Mutated (WT) (nd)*	31 (48) (1)
*TP53*	*Mutated (WT)*	31 (49)
Smoking history	*Never (current or former)*	9 (71)
Stage	*Ia/Ib*	44
	*IIa/IIB*	17
	*IIIa*	17
	*nd*	2

Number of samples not mutated, wild type (WT), are shown in brackets. No data, nd.

### Reverse Phase Protein Arrays

We have performed profiling of 295 cancer relevant proteins of which 60 were in a phosphorylated state (**Supplementary Table S2** and **Supplementary Material and Methods**) on the Oslo cohort, using the reverse phase protein array (RPPA) core facility at MD Anderson Cancer Center (Houston, TX). RPPA data (n=131 proteins) and phenotypes from the lung adenocarcinoma (LUAD) cohort were extracted from The Cancer Genome Atlas (TCGA) data generated by the TCGA Research Network: http://cancergenome.nih.gov/. Samples with no expression subtype assigned and without registered relapse free survival were filtered out. The remaining 181 LUAD samples were utilized as validation set. Time to relapse was extracted for survival analyses. Of note, the median follow-up time for alive patients was 23.9 months for the LUAD cohort, compared to 60 months for the Oslo cohort.

### EGFR, TP53, and KRAS Analyses

Mutation analyses of *EGFR* exons 18–21 were performed using the therascreen EGFR mutation kit (DxS, Manchester, UK) designed to detect 28 specific mutations in the *EGFR* gene. Assays were carried out according to the manufacturer’s protocol and with the use of the Roche LightCycler 480 Real-Time PCR System. Some of the results were previously published by Helland et al. (10).

The *TP53* gene was analyzed by the Sanger sequencing method in all the tumor samples. The procedure was performed on an Applied Biosystems 3730 DNA Analyzer according to the supplier’s handbook, Applied Biosystem 3730/3730X/DNA Analyzers Part 4331467 Rev.B, as previously described (11). More details are provided in **Supplementary Material and Methods**.

We used the wobble-enhanced ARMS (WE-ARMS) method for detecting *KRAS* mutations in the lung adenocarcinoma samples. This mutation assay detects the seven most commonly reported mutations in the *KRAS* gene—KRAS g.34G>C (p.G12R), g.34G>A (p.G12S), g.34G>T (p.G12C), g.35G>A (p.G12D), g.35G>C (p.G12A), g.35G>T (p.G12V), and g.38G>A (p.G13D)—by real-time PCR (12).

### Gene Expression and Subtyping of Adenocarcinoma Samples

Gene expression was performed on 186 adenocarcinomas (including 79 of those with protein expression) from the same cohort, using hybridization arrays (SurePrint G3 Human, 8x60K, Agilent Technologies). More details are provided in **Supplementary Material and Methods**. Clinical information such as pathological stage, mutation status, and smoking status are outlined in **Supplementary Table S1**. The adenocarcinoma samples were assigned a gene expression subtype using the previously described 506 gene centroid classifier and Pearson correlation (7, 8). Four of the samples had a negative correlation with all three subtypes and were not assigned to any subtype. Out of the 186 samples with gene expression, 14 were excluded in the Spearman’s test due to dead of other reasons than lung cancer. The data are deposited at ArrayExpress with accession number: E-MTAB-7954.

### Immunohistochemistry

Immunohistochemistry (IHC) staining for the neuroendocrine markers CD56, synaptophysin, chromogranin A, and NSE were performed on a selected subset of 11 samples, using the antibodies as outlined in **Supplementary Table S3** and **Supplementary Material and Methods**.

### Statistics

All the statistical analysis were performed in R (v 3.3.2) (13) using RStudio (v 1.1.447). Hierarchical clustering was performed to visualize the total protein expression for all the samples with mutation status, stage, subtype, smoking history, and relapse free survival (RFS) data included. Hierarchical clustering was performed using the R-package Clustermap (Lingjærde *et al.* personal communication). In brief, median-centered and log2-transformed RPPA-data were clustered using Pearson distance and average linkage, and with N=200 iterations in the PART method used to estimate the number of clusters, as described previously (14). For heatmap visualization of the data, values are normalized to the range [−1.2, 1.2] by application of a non-linear sigmoid transformation f(x) = 1.2*tanh(x/1.2). This limits the visual dominance of outlier values while maintaining the order of the values, since f is strictly increasing. RFS was calculated from the date of surgery until the date of event, defined as local relapse, metastases or death from lung cancer. Cut-off was set to 60 months. Correlation between protein expression/gene expression and RFS was calculated using two-sided Spearman’s test. Due to the restricted number of patients in this cohort, the p-value for these analyses are unadjusted. The reproducibility was assessed using bootstrapping method. Patients who died from other causes than lung cancer were filtered out in the Spearman’s test. Student t-test was performed to identify differentially expressed proteins between the groups of interests. Survival analysis was performed using cox regression analysis and Kaplan Meyer survival plot. The significance threshold was set to p<0.05. In order to reduce the false discovery rate, the p-value was adjusted using the Benjamini-Hochberg procedure.

## Results

We performed RPPA on biopsies from 80 lung adenocarcinoma biopsies. The tumor samples were tested for mutations in the *EGFR-*, *TP53-*, and *KRAS* genes, where 12.5% had *EGFR* mutations, 39% had *KRAS* mutations and 39% had mutations in the *TP53* gene. In the group of patients with *EGFR* mutated tumors, four (representing 44%) were never smokers (**Table 1**). Out of 80 patients, 36 (45%) patients were registered with RFS > 60 months. Among these, three (8%) of the tumors were *EGFR* mutated, 15 (42%) were *TP53* mutated, 13 (36%) were *KRAS* mutated and three patients were never smokers.

### Hierarchical Clustering Analysis

**Figure 1:**
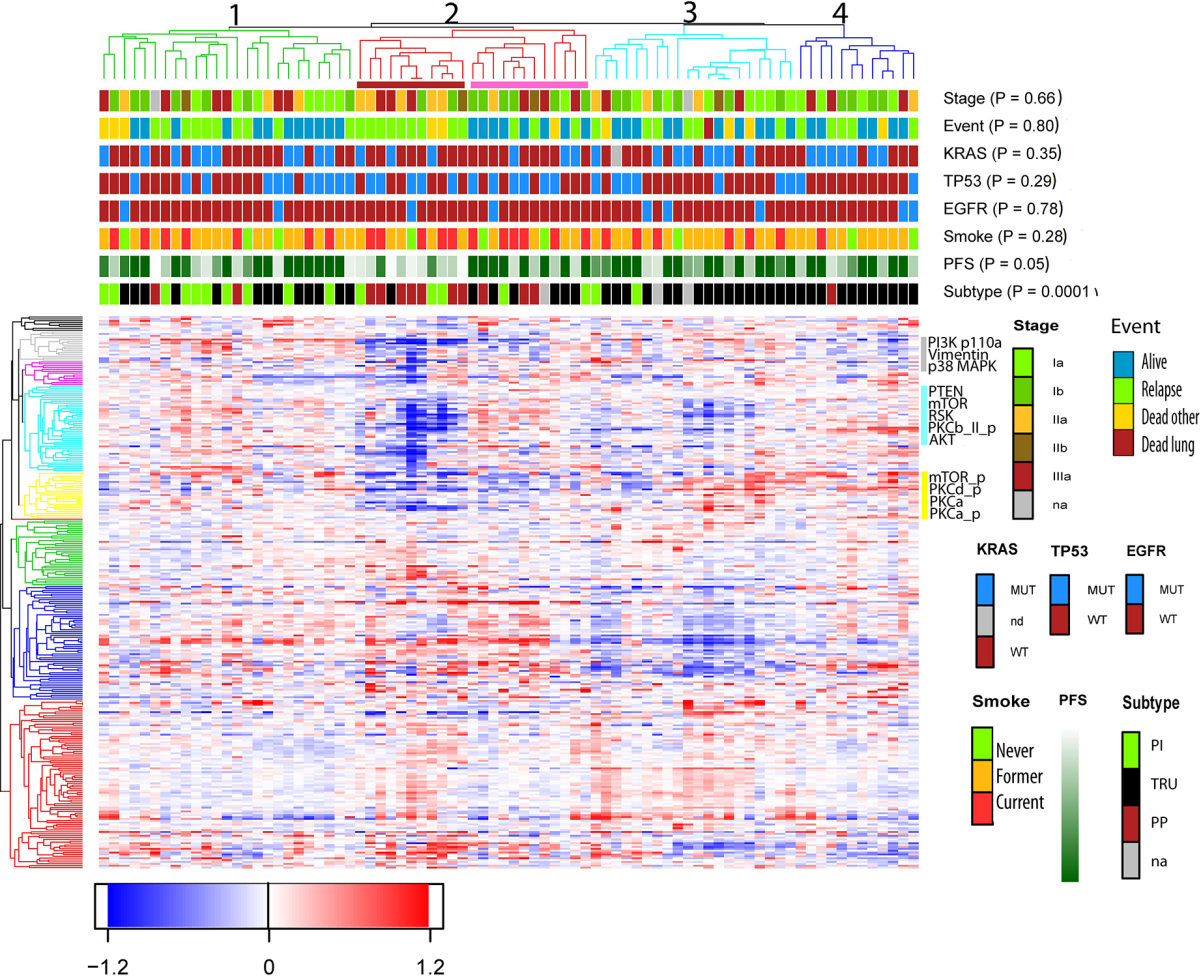
Unsupervised hierarchical cluster analysis based on protein expression from 80 non-small cell lung cancer (NSCLC) samples. Clinical variables such as smoking status, mutations status of the gene TP53, KRAS, and EGFR, RFS (ranging from 1 month = light green to dark green = 60 months) and event, were included to see if these features were enriched within the clusters. Events were divided into four categories; no event, relapse (which also includes metastasis), dead of lung cancer and dead of other reasons. The samples clustered into four subclusters marked with green, red, turquoise, and blue branches. Beneath subcluster two (red branch) a red box indicates a smaller cluster named 2a, and a pink box is drawn beneath subcluster 2b, 2c, and 2d. Subcluster 2a is recognized with poor RFS and enriched with subtype PP. The proteins are clustered into nine subclusters seen on the left side. The proteins PKCα, PKCα_p, and PKCδ_p belongs to the yellow cluster, and PKCβ-II_p is shown in the turquoise cluster.

**Figure 2:**
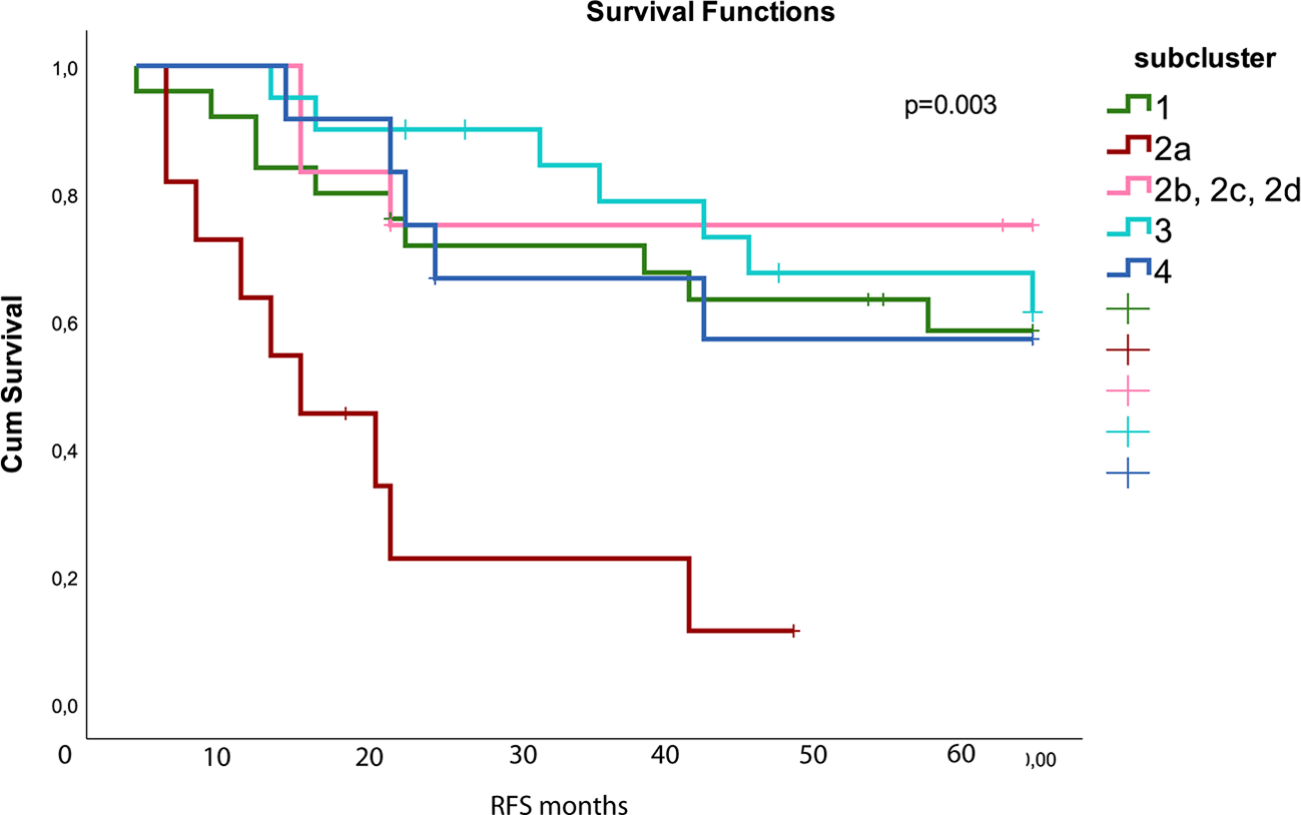
Kaplan Meier survival plot shows that subcluster 2a (red) had worse relapse free survival (RFS) compared to the other subclusters (p=0.003). The different subclusters are generated from the hierarchical clustering in **Figure 1**. Subcluster 2b contains 2b, 2c, and 2d shown with pink color. The colors used for the subclusters correspond to the color used for the subclusters in **Figure 1**. Number of samples in the different subclusters are: subcluster 1 n = 25, 2a: n=11, 2b/2c/2d: n = 12, 3: n = 20, and 4: n=12.

Unsupervised hierarchical clustering analysis performed on protein expression grouped the samples into four subclusters as shown in the dendrogram (**Figure 1**). The parameters *EGFR*-, *TP53*-, *KRAS*- mutations, stage, event, and smoking status were evenly distributed between the sub-clusters (p> 0.05 in Fisher exact test). The molecular subtypes were significantly associated with the four subclusters (p = 0.0001), of which subcluster 1 (green branches) was mainly associated with the PI subtype, subcluster 2 (red branches) enriched with the PP subtype, and subcluster 3 and 4 (turquoise and blue branches) were associated with the TRU subtype. Subcluster 2 contained four smaller clusters (2a, 2b, 2c, and 2d visualized in **Supplementary Figure S1**) of which 2a (marked with red box beneath of the branches in **Figure 1** and **Supplementary Figure S1**) was recognized with early relapse (median RFS = 9 months). We used multivariate cox regression analysis, where the samples in subcluster 2b, 2c, and 2d were analyzed as one group and stage was included as a covariate in the model, confirming a significantly shorter RFS for samples within subcluster 2a compared to the other subclusters (p=0.003). This association was visualized with a Kaplan Meier survival plot (**Figure 2**). The heatmap further demonstrated that cluster 2a had very low expression of several proteins recognized as members in the MAPK pathway and mTOR pathway. In addition, protein kinase C (PKC)-α and phosphorylated PKC-α- pS657, PKC-β-II- pS660, and PKC-δ- pS664 were significantly lower expressed in subcluster 2a compared to the other samples (Wilcoxon rank test, FDR <0.05, **Supplementary Table S4**). To identify the proteins with the highest correlation to RFS within this group, Spearman’s rank correlation was utilized (two samples reported with non-lung cancer related death were excluded in this analysis). Six proteins (myosin11, CD26, caspase 7 cleaved, YAP, ER, and 4E-BP1-pT37-T46) were significantly associated (p< 0.05) with RFS in subcluster 2a (**Supplementary Table S5**). To check if subcluster 2a contained neuroendocrine-like features, the expression of the genes *NCAM1*, *CHGA*, *SYP* encoding for the neuroendocrine markers CD56, chromogranin A, and synaptophysin respectively, were analyzed. All three markers revealed a higher gene expression in samples from subcluster 2a compared to all the other samples, but only *SYP* was significant (p=0.03). IHC staining for the proteins CD56, chromogranin A, synaptophysin, and neuron-specific enolase (NSE) were performed for the 11 samples in subcluster 2a, and confirmed an positive expression of synaptophysin, CD56, chromogranin A, and NSE in seven, one, one, and nine of the 11 samples, respectively (**Supplementary Table S6**). Serum levels of the neuroendocrine markers proGRP, chromoganin A, and NSE were elevated in one of the 11 samples (data not shown).

Unsupervised hierarchical clustering of mRNA expression data of the genes coding for the analyzed proteins (n=226) were performed on a cohort of 186 adenocarcinoma samples, including the 79 with RPPA data (**Supplementary Figure S2**). The clustering confirmed a different gene expression pattern for the three molecular subtypes (p = 1.3*E−16 in Fisher exact test). The subtype PI was scattered between several subclusters, but the majority of the TRU samples were found in the largest subcluster, whereas most of the PP subtype samples clustered together. Focusing on the 11 samples from subcluster 2a identified using protein expression data, only the samples with the PP subtype (n=7) clustered together with samples recognized with poor RFS and PP subtype, when using mRNA data. Time to progression, *TP53* - and *EGFR*- mutation status were significantly differentially distributed between the subclusters (p < 0.05).

### Protein Expression Associated With Prognosis

Using Spearman’s rank correlation, we identified 46 proteins significantly associated with RFS (unadjusted p<0.05), of which eight were in a phosphorylated state (**Supplementary Table S5**). We used bootstrapping (n=1,000 bootstrapping samples) to evaluate the standard deviation of the rho value; 45 of the 46 proteins reported with p-value <0.05 had rho-values within two standard deviations, including all PKC proteins, confirming the reproducibility of the test (**Supplementary Table S7**). High expression of PKC-α, and phosphorylated PKC-α, PKC-βII, and PKC-δ were all highly associated with increased RFS as outlined in **Table 2** (p<0.05).

**Table 2:** Top 10 proteins correlated with RFS (Spearman’s rank correlation, p<0.05).

Protein	Rho	p-value	Adjusted p-value	rho.sd	2.sd	rho>2*SD
c.Abl	−0.422	<0.001	0.081	0.102	0.204	Yes
PKC.b.II_pS660	0.376	0.001	0.141	0.11	0.22	Yes
PAI.1	−0.372	0.002	0.141	0.107	0.214	Yes
PKC.a	0.356	0.002	0.141	0.107	0.214	Yes
MIF	−0.346	0.003	0.141	0.11	0.22	Yes
PKC.a_pS657	0.35	0.003	0.141	0.108	0.216	Yes
PKC.delta_pS664	0.348	0.003	0.141	0.119	0.238	Yes
LC3A.B	−0.338	0.004	0.155	0.12	0.24	Yes
Caveolin.1	0.323	0.006	0.165	0.109	0.218	Yes
GPBB	0.323	0.006	0.165	0.121	0.242	Yes

The total list of the 46 identified proteins from this analysis including adjusted p-value (Benjamini-Hochberg) is displayed in **Supplementary Table S7**. The correlation coefficient shows the strength and direction between the two variables RFS and protein expression, and bootstrapping was utilized to evaluate the standard deviation of the rho value.

Spearman’s rank correlation performed pair-wise for 79 samples on the proteins and phosphorylated proteins, and the corresponding gene expression data (**Supplementary Figure S3**, **Supplementary Table S8**) revealed a positive correlation (>0.3) for 110 of the proteins, and a negative correlation for 42 of the proteins.

Next, we included the samples with mRNA data (n=172) and performed a Spearman’s Rank correlation between genes and proteins associated with prognosis, and found 13proteins/genes significantly associated with RFS in both analysis (**Table 3** and **Supplementary Figure S3**).

**Table 3:** Intersection of messenger RNAs (mRNAs) and proteins significantly correlated to relapse free survival (RFS).

Pair-wise correlation mRNA/protein			mRNA significantly correlated to RFS	Protein significantly correlated to RFS
Gene name	Protein name	Spearman’s rho	Spearman’s rho	Spearman’s rho
AR	AR	0.475	0.172	0.241
CCNB1	Cyclin.B1	0.758	−0.256	−0.257
CCNE1	Cyclin.E1	0.682	−0.177	−0.254
DPP4	CD26	0.720	0.274	0.266
KIT	c.Kit	0.815	−0.232	−0.253
LCK	Lck	0.615	0.172	0.287
MIF	MIF	0.549	−0.252	−0.346
PIK3R1	PI3K.p85	0.38	0.193	0.264
PRKCA	PKC.a	0.648	0.208	0.356
PRKCD	PKC.delta_pS664	0.18	0.225	0.348
RPS6KA1	RSK	0.543	0.244	0.272
SLC1A5	SLC1A5	0.520	−0.184	−0.263
STAT5A	Stat5a	0.462	0.228	0.265

The pair-wise Spearman’s rank correlation mRNA/protein was performed on 79 samples. Only significantly positively pairwise correlations and mRNA/proteins simultaneously significantly correlated to RFS are included in the list. In the RFS analysis, patients reported with non-lung cancer related death were filtered out.

### Protein Expression With Impact on Prognosis Differ Depending on Mutational and Smoking Status

A significant association between RFS and expression of 15 proteins (**Supplementary Table S5**), including MEK-1, phosphorylated bad, collagen VI, LCA3B, and CD49b was seen in never smokers. Two proteins (LCA3B and Myosin 11) were significantly associated with RFS in both never-smokers and smokers (**Supplementary Figure S4**).

High expression of B7-H3 was associated with poor survival in the total group, but only found to be significant in *KRAS* and *TP53* wild type tumors, smokers, and *EGFR* mutated samples after stratification (p<0.05, **Supplementary Table S5**). Seven out of the nine EGFR mutated samples showed B7-H3 expression level above the average in the total group.

When comparing similarities in RFS-associated proteins between the mutated samples and the wild type samples one, three and four overlapping proteins were identified in the EGFR, KRAS, and TP53 subgroups, respectively. The proteins PKC-α and phosphorylated PKC-α, PKC-βII, and PKC-δ were the most significantly RFS-associated proteins in smokers, *TP53*wt, *EGFR*wt, and *KRAS*wt. Samples with EGFRmut, RFS was significantly associated with PKC-δ, where five out nine samples showed expression level above average in the total group. In samples with TP53mut or KRASmut, RFS was significantly associated with PKC-α and phosphorylated PKC-α. Among the mutated samples, fewer proteins were associated with RFS, and as expected, no overlapping proteins were found between *EGFR* and *KRAS* mutated samples (**Supplementary Table S5** and **Supplementary Figure S4**). The same analyses were applied to the 172 samples with gene expression data, where fewer genes were associated with RFS in samples harboring a mutation compared to wild type samples. In the groups stratified on mutational status and smoking status, the level of *PRKCA*, encoding PKC-α, were significantly associated with RFS in smokers, *EGFR* wt, *KRAS* wt, and *TP53* mut samples. The level of *PRKCD*, encoding PKC-δ, was significantly associated with RFS among smokers, *EGFR* wt, *EGFR* mut, *KRAS* wt, and *TP53* wt.

**Figure 3:**
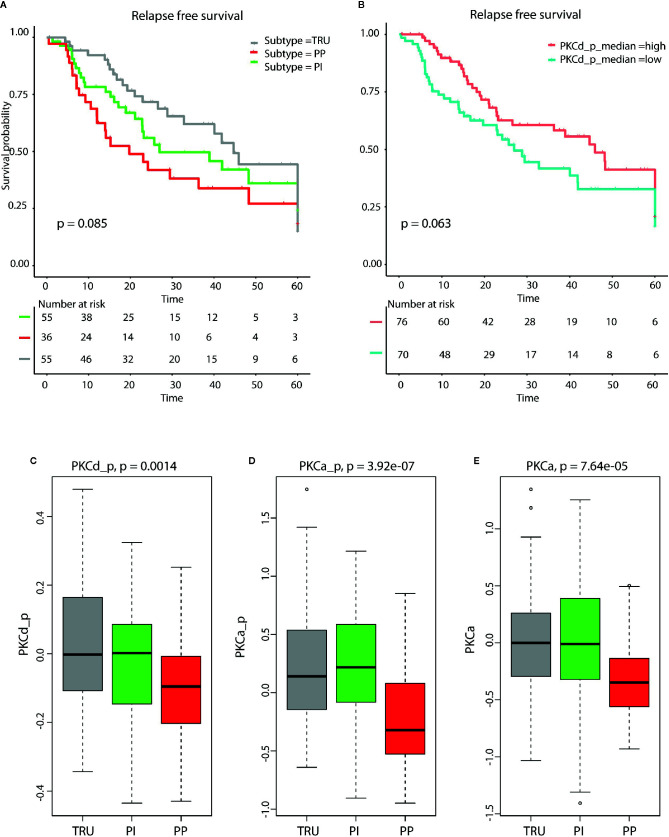
**(A)** Kaplan Meyer survival plot demonstrate reduced relapse free survival in patients with PP subtype (p = 0.085). **(B)** Kaplan Meyer survival plot shows that low levels of phosphorylated PKCd (p= 0.0683, log rank test) are associated with reduced relapse free survival. The molecular subtypes terminal respiratory unit (TRU), proximal inflammatory (PI), and proximal proliferative (PP) display different levels of **(C)** phosphorylated PKCδ, **(D)** phosphorylated PKCα, and **(E)** PKCα. The lowest level is seen in the PP samples for all three proteins. These results are calculated from lung adenocarcinoma (LUAD) samples in The Cancer Genome Atlas (TCGA).

A tendency toward worse relapse free survival in patients with low expression of PKC-δ was also seen in the TCGA data (p=0.06, **Figure 3**). The PP subtype showed a significantly decreased expression of PKC-α, and phosphorylated PKC-α and PKC-δ when compared to PI and TRUE subtype. Further, the PP subtype also showed a reduced relapse free survival when compared to non-PP samples (p=0.085, **Figure 3**).

## Discussion

In this study we examined the expression of 235 proteins and 60 phospho-proteins in tumors from 80 patients diagnosed with lung adenocarcinomas. We identified 46 proteins and phospho-proteins likely to impact the patient outcome. When stratifying the samples according to mutations in the genes *TP53*, *EGFR*, or *KRAS*, more proteins were associated with RFS in samples without mutations. This was also seen when analyzing the corresponding mRNA data. Cluster analysis identified a small subcluster containing 11 patients enriched with tumors of the PP subtype, recognized with early relapse, low expression of PKC isozymes, and increased expression of neuroendocrine markers.

### Cluster Analysis

Based on the expression of 295 proteins, unsupervised hierarchical clustering separated the samples into four subclusters. However, pathological stage, smoking status, or mutations in the genes *TP53*, *KRAS* or *EGFR* did not seem to impact the clustering. Interestingly, the four subclusters were significantly correlated with the molecular subtypes TRU, PP, and PI. Previous work using protein expression identified six subgroups of adenocarcinoma, where the subgroups partially overlapped with the three mRNA-derived subtypes. The PP subtype was further divided into two groups (9), which also was seen in our data set. Subcluster 2a, enriched with PP subtype samples, was recognized with lower expression of members of the mTOR pathway and the MAPK pathway. This is in line with previous finding where TRU samples are associated with higher expression of these proteins when compared to PP samples (15). Interestingly, cluster analysis performed on mRNAs corresponding to the proteins, grouped the samples with PP subtype from the protein-derived subcluster 2a together, but not the non-PP subtypes. However, the distinct pattern with very low expressed proteins within subcluster 2a, was not reflected using mRNA data. This can be explained with a lower correlation between phosphorylated proteins and the corresponding genes. Seven of the 11 samples within subcluster 2a showed a positive staining for synaptophysin which are considered as a neuroendocrine marker and can be used to confirm the diagnosis (16). In total, 10 of the 11 samples showed positive staining for at least one of the neuroendocrine marker or NSE. According to WHO 2015, about 10–20% of lung tumors lacking neuroendocrine morphology by light microscopy may reveal neuroendocrine differentiation using IHC (17). In a recent study on NSCLCs, a molecular subgroup enriched with PP subtype and shorter survival was identified. These samples had a mixed histology predominantly with adenocarcinomas with molecular expression pattern associated with neuroendocrine tumors (15). LCNEC (large cell neuroendocrine carcinoma) can share some of the same pathological features as adenocarcinomas, and LCNEC with areas of adenomatous differentiations (mixed LCNEC) is described. Both pure LCNEC and mixed LCNEC tumors exhibit an aggressive behavior and are associated with poor survival (18) as seen within subcluster 2a in our analyses.

The hierarchical clustering performed on 186 mRNA samples resulted in a significantly different distribution of the molecular subtypes between the clusters, although the PI samples did not cluster together. Of note, the overlap between the genes used for the original sub-typing (8) and our clustering based on protein expression was sparse (n=18), indicating that the proteins included in our analysis seem to be important for the subtypes. Both the TRU- and PP-subtype clustered based on our mRNA data, and we suggest that the TRU- and PP-subtypes are more distinct subtypes compared to PI. In a large meta-study, the TRU subtype was identified as the most prognostically important subtype compared to the non-TRU subtype, arguing for the need to identify additional classifiers (19).

### Protein Kinase C Levels Associated With Survival

The protein kinase C is a group of enzymes known to be involved in diverse cellular functions, including cell proliferation, apoptosis, and cell migration, and has been regarded as an onco-protein. The members of the PKC-family are encoded from nine different genes which have several known splice variants. Recent work has demonstrated that these proteins may have a more complex role than first assumed, which is supported by the many failed clinical trials for cancer using PKC-inhibitors. In addition, mutational studies have revealed that most cancers have loss of function (LOF) mutations in genes belonging to the PKC-family, suggesting a tumor suppressor role for the proteins (20, 21). A meta-study on the use of PKC-inhibitors combined with chemotherapy in lung cancer patients reported decreased response rate and disease control, compared to chemotherapy alone (22). In our study, low expression of PKC-α, and phosphorylated PKC-α, PKC-βII, and PKC-δ were associated with poor RFS. In addition, the levels of the PKC isozymes were strikingly lower in subcluster 2a which also contained the samples with the overall poorest RFS. An association with low expression of PKC- δ and decreased relapse free survival was confirmed in the LUAD TCGA samples. Low levels of PKC-α, and phosphorylated PKC-α and PKC-δ in subtype PP were also found in the TCGA samples. This support the findings in the Oslo cohort, with poor RFS and low expression of the isozymes of PKC in subgroup 2a containing mainly PP samples. Unfortunately, sparse information on mutational status, reduced number of proteins analyzed, short follow-up time, limited further validation on the TCGA samples.

Thus, with regard to the classical role of the PKC-family, our results suggest a general tendency toward a tumor suppressor role for PKC-α, PKC-βII, and PKC-δ in NSCLC.

In the *EGFR* mutated samples, PKC-δ levels positively correlated to patient survival. It has been reported that an activation of PKC-δ can be promoted by an activated EGFR (23). Further, activation of PKC-δ may induce apoptosis and growth arrest resulting in reduced tumorigenesis (24). As a response to DNA damage, it has been shown that over-expression of p53 increases the transcription of PKC-δ resulting in apoptosis. This may explain the significant correlation we discovered between the protein expression of PKC-δ and RFS in *TP53* wild type samples, but not in the *TP53* mutated samples. Nevertheless, this was not reflected by the mRNA analyses. It’s been demonstrated that PKC can phosphorylate many oncoproteins to suppress their activity, including KRas, PI3K, and several tyrosine kinase receptors (25). The oncoprotein KRas, recognized with activating mutations in cancer, can be suppressed by activated PKC, which is proposed as a novel approach to target KRas (26). A negative correlation between KRas and PKC-α was recently described in colorectal cancer. Further, low expression of PKC-α was also associated with poor prognosis (27). This is in concordance with our results, where high levels of both PKC-α and PKC-δ was associated with better RFS. The level of PKC-δ showed a higher correlation to RFS in those with *KRAS* wild type, which also were confirmed when using mRNA data. Interestingly, no isozymes of PKC did influence on survival in never-smoking patients, further supporting this group as a distinct lung cancer disease driven by other mechanisms.

This leads to the hypothesis that PKC may also have an essential role keeping oncoproteins in check (25). Based on our results, we suggest that the association to RFS for the different PKC- isozymes is connected to mutational and smoking status. Results from gene expression analysis performed on 172 NSCLC samples strengthen these observations. Limitation to this study is the small number of samples included in the protein analysis, and the lack of a proper validation material. Due to the restricted number of patients in this cohort, the p-value for Spearman’s rank correlations analyses are unadjusted. However, we included the bootstrapping method, which confirmed the reproducibility of our results. More analyses are warranted in order to confirm our findings. Unfortunately, we have not found similar RPPA studies in lung cancer tissue for comparison with our results.

### Proteins Associated With Relapse Free Survival in Subgroups of Non-Small Cell Lung Cancer

Interestingly, high expression of B7-H3, a molecule involved in immune checkpoint signaling, was correlated to poor outcome in our study, especially in smokers, those without any detected mutations in *KRAS* or *TP53*, and in those harboring an *EGFR* mutation. B7-H3 is a molecule known to inhibit T-cell activation in an immune suppressive manner. This protein has been shown to be linked to poor survival in cancer, and have been suggested as a new immune checkpoint target (28). In a recent study of lung cancer patients, expression of B7-H3 was associated with overall survival only in smokers (29). This indicates that future anti-B7-H3 therapy may have higher success rate among ever smoking lung cancer patients with *KRAS* or *TP53* wild type tumors, or an *EGFR* mutation.

Within subcluster 2a, six proteins were significantly associated with RFS, where low expression of myosin II showed the highest correlation with better RFS. This is also supported by a protein study on early stage lung cancer where myosin IIa was reported to be upregulated in stage Ia/Ib lung cancer patients with early relapse (30). Interestingly, low expression of YAP was significantly associated with increased RFS in subcluster 2a. Further, a tendency toward better RFS was associated with low expression of phosphorylated YAP (p=0.1) and phosphorylated HSP27 (p=0.12). It has been shown that high expression of HSP27 leads to less phosphorylated YAP (s127). Further, phosphorylation of YAP on S127 decreased the activity of YAP since this prevent its translocation to the nucleus (31). This also means that un-phosphorylated YAP promotes tumor aggressiveness and is related to poor prognosis which is in line with our study. These finding highlight the central role HSP27 has in several pathways, including the Hippo pathway.

### Correlation Analysis Between Messenger RNA and Protein Expression

Spearman’s Rank correlation revealed a high correlation (rho >0.3) between expression levels of almost half of the proteins and mRNAs. Previous studies have reported that much of the variation in mean-level protein expression can be explained by variation in mRNA expression (32, 33). However, the variance in the proteomes across different tissue types can poorly be explained by the mRNA levels, highlighting a tissue-specific posttranscriptional regulation of gene expression. In a study of lung cancer, proteins involved in metabolic and translational pathways were highly correlated with mRNA expression, whereas proteins involved in extracellular matrix and adhesion, were not correlated or anti-correlated (33). In a study of breast cancer, 35% of the proteins correlated significantly (rho > 0.3) with mRNA expression. The proteins, cyclin B1, cyclin E1, 4E-BP1, PKC-α, and RAB 25 were highly correlated with mRNA expression in breast cancer (34). This is in line with our study, where these proteins showed a high correlation value (rho > 0.6). Interestingly, HER2 was highly correlated in the breast cancer study across all subtypes, but this protein was poorly correlated with mRNA expression in our lung study (rho =0.17). On the other hand, EGFR revealed rho=0.72 in our study, while in breast cancer a correlation between rho=0.15–0.3 was found. This indicates that genes known to be deregulated in a specific cancer type may be regulated by other mechanisms. Proteins such as p53, CDKN1B, and MAPK14 showed very low correlation with the mRNA expression both in our study on lung cancer and in the breast cancer study (34). Lack of correlation between the level of proteins and mRNAs measured in the cells can have several explanations including copy number aberrations, miRNA expression, and methylation.

## Conclusion

These results demonstrate that essential mutations in lung carcinomas affect several proteins associated with outcome. Based on our results, expression of PKCα and phosphorylated PKCα, PKCβ, and PKCδ seem to be positively associated with RFS, with different isozymes linked to smoking and mutational status of *EGFR*, *KRAS*, and *TP53*. These results illustrate the need to better understand the biological context in order to further improve targeted therapy in cancer. This study supports that a therapy restoring the level of specific isozymes of PKC activity may be beneficial for subgroups of lung cancer patients based on the genetic background. However, further studies exploring these findings are needed. We identified a subgroup of samples enriched with the molecular subtype PP, recognized with early relapse, increased expression of neuroendocrine markers, and a distinct protein expression pattern, including low levels of PKC isozymes. These patients may benefit from a more aggressive treatment regimen. Proteins associated with RFS among never smokers were strikingly different compared to the other investigated subgroups. This is not surprising, but underscores the need for a more stratified therapy in order to improve clinical outcome.

## Data Availability Statement

The gene expression data are deposited at Array Express with accession number: E-MTAB-7954. Protein expression data are listed in **Supplementary Table 2**.

## Ethics Statement

The study was approved by the Regional Ethics Committee (S-05307). The patients/participants provided their written informed consent to participate in this study.

## Author Contributions

MH, ÅH, and ARH conceived and designed the project. ARH and ÅÕ acquired the data. ARH, MH, ÅÕ, DN, GM, and ML-I analyzed and interpreted the data. ARH, ÅH, and MH wrote the paper. SS, LJ, ÅH, and OB collected the clinical data and the material. All authors reviewed the data analyses. All authors contributed to the article and approved the submitted version.

## Funding

This work was economically supported by the The Norwegian Cancer Society [grant number 88503-2013]; and south-Eastern Norway Regional Health Authority [grant number 2016056]. The RPPA analysis was provided by the Sister Institution Network Fund (SINF) in FY14 to Prof. Gordon Mills and the RPPA core facility at the MD Anderson Cancer Center (MDACC CCSG grant P30 CA016672 and NCI # CA16672). The funding bodies have not influenced the design of the study, the collection of data, the analyses or the interpretation of data.

## Conflict of Interest

The authors declare that the research was conducted in the absence of any commercial or financial relationships that could be construed as a potential conflict of interest.

## Supplementary Material

Supplementary Figure 1 Hierarchical clustering of subcluster 2 derived from Fig 1.

Supplementary Figure 2 Hierarchical clustering of genes corresponding to the proteins included in RPPA. Clinical variables such as smoking status, mutations status of the gene TP53, KRAS and EGFR, RFS (ranging from 1 month = light green to dark green = 60 months) and event, were included to see if these features were enriched within the clusters. Events were divided into four categories; no event, relapse (which also includes metastasis), dead of lung cancer and dead of other reasons.

Supplementary Figure 3 Waterfall plot shows the correlation between protein expression (included proteins in phosphorylated state) and mRNA expression. Y-axis displays the Spearman’s rho coefficient. The proteins/genes are distributed on x-axis ordered after degree of correlation. The 13 overlapping RFS associated proteins/genes are displayed with black spikes.

Supplementary Figure 4 Proteins associated with RFS in groups stratified on mutational status and smoking status. Cases recorded with dead of other reasons are filtered out.

## Abbreviations

ALK, anaplastic lymphoma receptor tyrosine kinase; CIN, chromosomal instability; EGFR, epidermal growth factor receptor; ERK, extracellular signal-regulated kinase; IHC, Immunhistochemistry; KRAS, Kirsten rat sarcoma viral oncogene homolog; LCNEC, large cell neuroendocrine carcinoma; LOF, loss of function; MEK, mitogen-activated extracellular signal-regulated kinase; NSCLC, non-small cell lung cancer; PI, proximal inflammatory; PKC, protein kinase C ; PKC_p, phosphorylated protein kinase C; PP, proximal proliferative; RFS, relapse free survival; RPPA, reverse phase protein array; TP53, tumor protein p53; TRU, terminal respiratory unit; WT, wild type.
