# Supplementary material for: Protein Kinase C Isozymes Associated With Relapse Free Survival in Non-Small Cell Lung Cancer Patients
Source: Front Oncol. 2020 Nov 25;10:590755. doi: 10.3389/fonc.2020.590755 (PMC7725872; doi:10.3389/fonc.2020.590755)
Supplement: Supplementary Figure 1 — Hierarchical clustering of subcluster 2 derived from Fig 1. [file DataSheet_1.pdf]

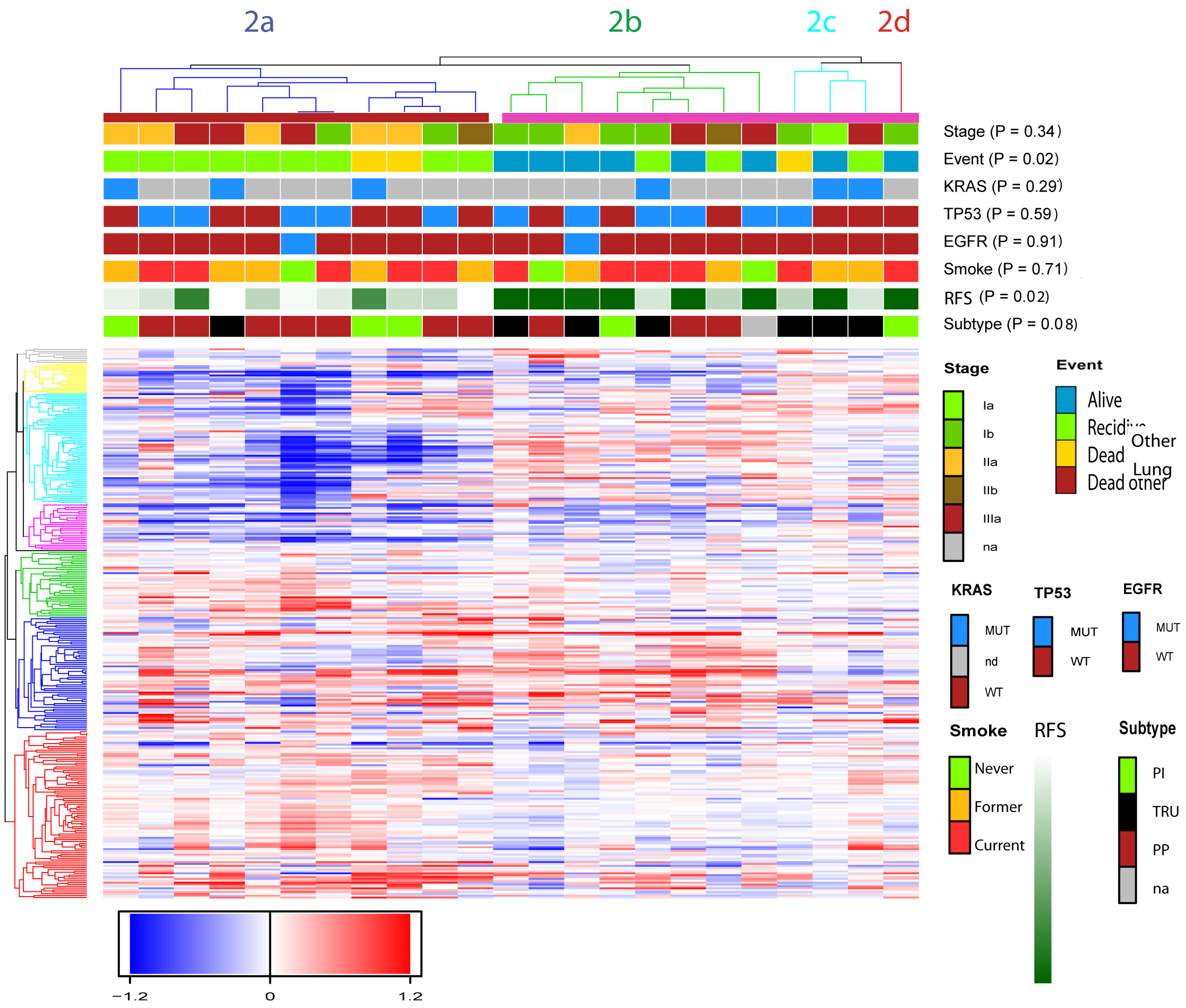
2a
2b
2c
2d
Stage (P = 0.34)
Event (P = 0.02)
KRAS (P = 0.29)
TP53 (P = 0.59)
EGFR (P = 0.91)
Smoke (P = 0.71)
RFS (P = 0.02)
Subtype (P = 0.08)
Stage
Ia
Ib
IIa
IIb
IIIa
na
Event
Alive
Recidive
Dead Other
Dead Lung
KRAS
MUT
nd
WT
TP53
MUT
WT
EGFR
MUT
WT
Smoke
Never
Former
Current
RFS
Subtype
PI
TRU
PP
na
−1.2
0
1.2
