# Supplementary figures and images for: Protein Kinase C Isozymes Associated With Relapse Free Survival in Non-Small Cell Lung Cancer Patients
Source: Front Oncol. 2020 Nov 25;10:590755. doi: 10.3389/fonc.2020.590755 (PMC7725872; doi:10.3389/fonc.2020.590755)

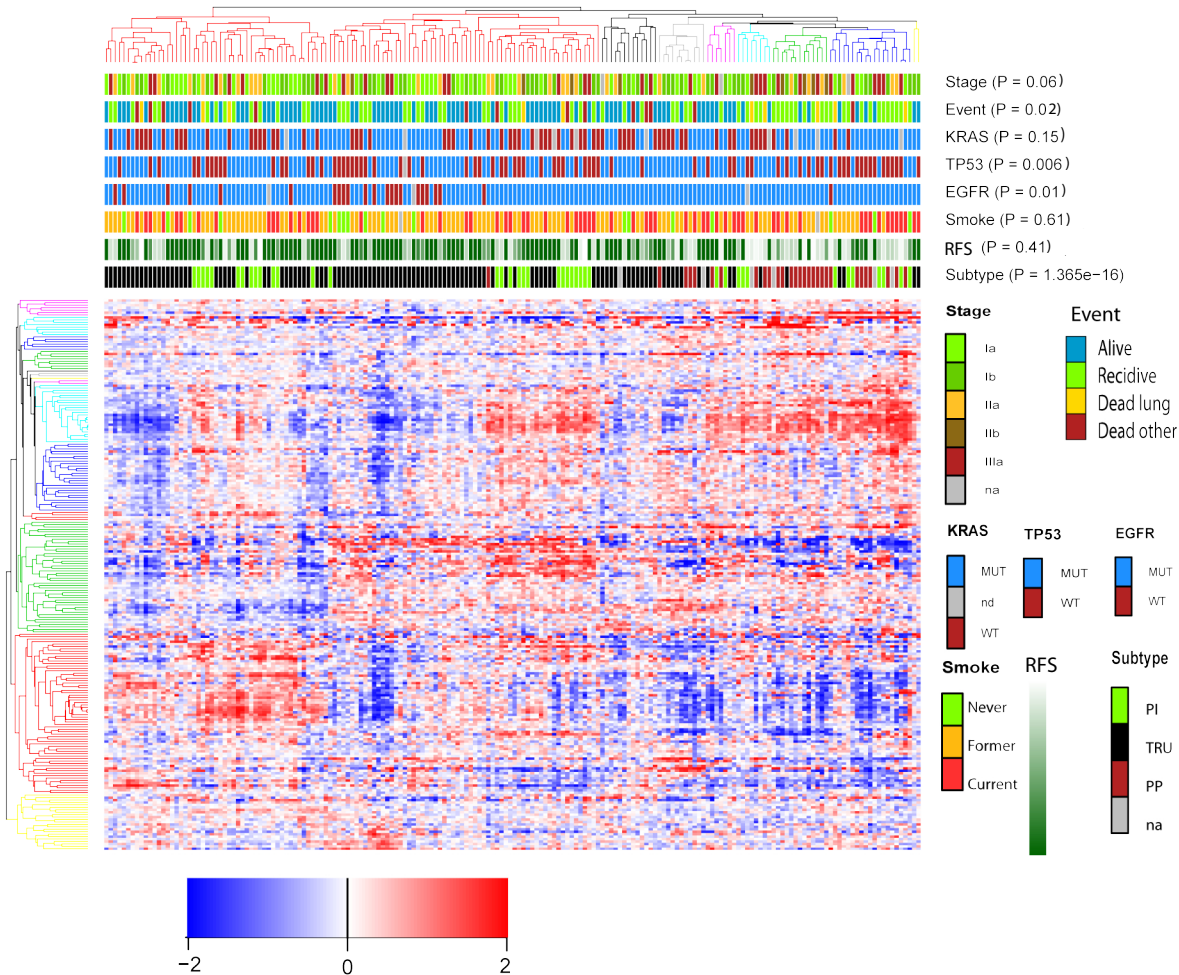

Supplement: Supplementary Figure 2 — Hierarchical clustering of genes corresponding to the proteins included in RPPA. Clinical variables such as smoking status, mutations status of the gene TP53, KRAS and EGFR, RFS (ranging from 1 month = light green to dark green = 60 months) and event, were included to see if these features were enriched within the clusters. Events were divided into four categories; no event, relapse (which also includes metastasis), dead of lung cancer and dead of other reasons. [file DataSheet_2.pdf]

Spearman's correlation between mRNAs and proteins/phosphorylated proteins

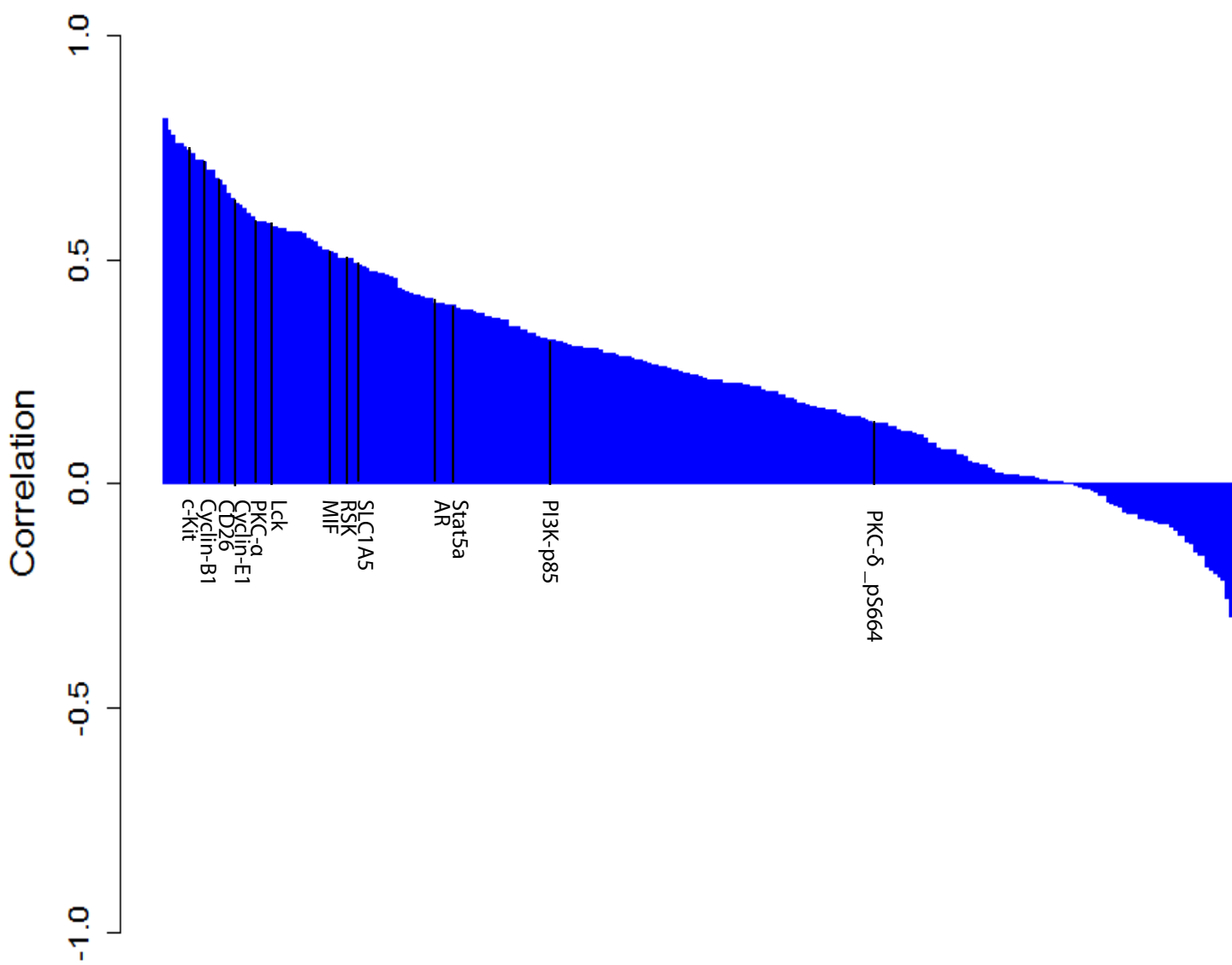

Supplement: Supplementary Figure 3 — Waterfall plot shows the correlation between protein expression (included proteins in phosphorylated state) and mRNA expression. Y-axis displays the Spearman’s rho coefficient. The proteins/genes are distributed on x-axis ordered after degree of correlation. The 13 overlapping RFS associated proteins/genes are displayed with black spikes. [file DataSheet_3.pdf]
