# Supplementary material for: Protein Kinase C Isozymes Associated With Relapse Free Survival in Non-Small Cell Lung Cancer Patients
Source: Front Oncol. 2020 Nov 25;10:590755. doi: 10.3389/fonc.2020.590755 (PMC7725872; doi:10.3389/fonc.2020.590755)
Supplement: Supplementary Figure 4 — Proteins associated with RFS in groups stratified on mutational status and smoking status. Cases recorded with dead of other reasons are filtered out. [file DataSheet_4.pdf]

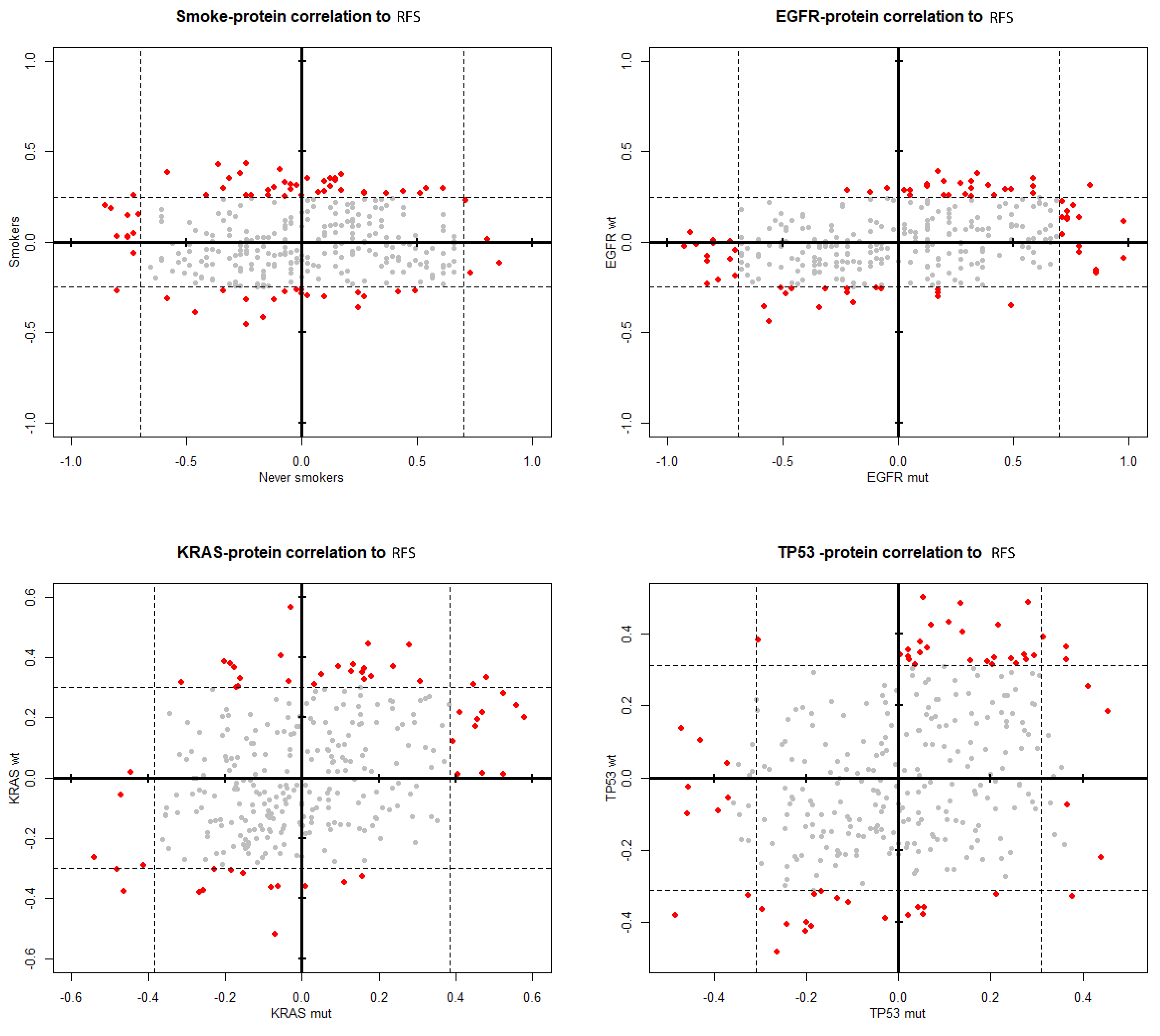

Smoke-protein correlation to RFS
Smokers
Never smokers
EGFR-protein correlation to RFS
EGFR wt
EGFR mut
KRAS-protein correlation to RFS
KRAS wt
KRAS mut
TP53 -protein correlation to RFS
TP53 wt
TP53 mut
