# Supplementary material for: Protein Kinase C Isozymes Associated With Relapse Free Survival in Non-Small Cell Lung Cancer Patients
Source: Front Oncol. 2020 Nov 25;10:590755. doi: 10.3389/fonc.2020.590755 (PMC7725872; doi:10.3389/fonc.2020.590755)
Supplement: Supplementary file 5 [file DataSheet_5.docx]

# Material and methods supplementary data

### Gene expression

Gene expression was performed on 186 adenocarcinomas (including 79 of those with protein expression) from the same cohort, using hybridization arrays (SurePrint G3 Human, 8x60K, Agilent Technologies). First total RNA (totRNA) was extracted from the tumor samples with standard Trizol protocol (Invitrogen, Carlsbad, CA). The yield and quality of the RNA was determined with Nanodrop ND-1000 spectrophotometer (NanoDrop technologies). The RNA integrin numbers (RIN) was assessed by 2100 Bioanalyzer (Agilent Technologies). First 50ng totRNA was reverse transcribed, amplified and labeled with cyanine 3-CTP. The labeled cRNA were hybridized to slides and incubated for 17 hours at 65°C. Arrays were scanned by using Agilent Microarray Scanner (Agilent technologies) and the raw data were preprocessed with the Agilent`s Feature Extraction Software with default parameters (Agilent feature extraction version 10.7.3.1). The gene expression data was log2 transformed and quantile normalized in Genespring GX analysis Software v.12.1 (Agilent Technology).

### EGFR, TP53 and KRAS analyses

Mutation analyses of EGFR exons 18–21 were performed using the TheraScreen EGFR mutation kit (DxS, Manchester, UK,) designed to detect 28 specific mutations in the EGFR gene. Assays were carried out according to the manufacturer’s protocol and with the use of the Roche LightCycler 480 real-time PCR system. Some of the results were previously published by Helland et al.

The TP53 gene was analyzed by the Sanger Sequencing method in all the tumor samples. The procedure was performed on an Applied Biosystems 3730 DNA analyser according to the supplier’s handbook, Applied Biosystem 3730/3730X/DNA Analysers Part 4331467 Rev.B, as previously described. All exons from 2 to 11, including the 16 flanking base pairs of each exon, were investigated. The sequences were aligned and analyzed using SeqScape v.2.5 according to the project template [TP53 accession nr: [NM_000546](https://www.ncbi.nlm.nih.gov/nuccore/NM_000546)^[4](https://www.ncbi.nlm.nih.gov/pmc/articles/PMC4863128/#fn04)^(TP53ref_NC000017.9_NT010718.15)]. All the sequences were manually and independently evaluated by two persons. Sequences harboring missense mutation, non-sense mutation, insertion or deletion in the *TP53* sequence were regarded as mutated.

We used the wobble-enhanced ARMS (WE-ARMS) method for detecting KRAS mutations in the lung adenocarcinoma samples. This mutation assay detects the seven most commonly reported mutations in the KRAS gene—KRAS g.34G>C (p.G12R), g.34G>A (p.G12S), g.34G>T (p.G12C), g.35G>A (p.G12D), g.35G>C (p.G12A), g.35G>T (p.G12V) and g.38G>A (p.G13D)—by real-time PCR.

### Reverse phase protein arrays

We have performed profiling of 295 cancer relevant proteins of which 60 were in a phosphorylated state (Table S1), using the reverse phase protein array (RPPA) core facility at MD Anderson Cancer Center (Houston, TX). Tumor protein lysates were serially diluted two-fold for 5 dilutions (from undiluted to 1:16 dilution) and arrayed on nitrocellulose-coated slides (in an 11x11 format). Samples were probed with antibodies by tyramide-based signal amplification approach, visualized by DAB colorimetric reaction. Slides were scanned on a flatbed scanner to produce 16-bit tiff image and the spot density was quantified by Array-Pro Analyzer. Relative protein levels for each sample were determined by interpolation of each dilution curves from the "standard curve" (SuperCurve Rx64 3.1.1) of the slide (antibody). All the data points were normalized for protein loading by using a protein loading correction factor (CF). Each sample has its unique correction factor. If the correction factor is less than 0.25 or greater than 2.5, we consider these samples “outliers,” indicating that protein concentration is much lower or much higher than the other samples. All the values were log2 transformed and median centered across each antibody.

### Immunohistochemistry (IHC)

Immunohistochemistry (IHC) was performed on whole section of surgical specimen to fulfill histological characterizations with visual assessment of the tumor morphology and cell structure. The proportion of the neuroendocrine markers CD56, synaptophysin, chromogranin A and neuron-specific enolase (NSE) within the tumor cells was evaluated. Slides were reviewed by a pathologist. Negative and positive controls were included. The samples were considered as positive if more than 1% of the cells were positive. The results are shown in Supplementary Table S5. The antibodies used for staining are listed in Supplementary Table S2.
