# Supplementary material for: Protein Kinase C Isozymes Associated With Relapse Free Survival in Non-Small Cell Lung Cancer Patients
Source: Front Oncol. 2020 Nov 25;10:590755. doi: 10.3389/fonc.2020.590755 (PMC7725872; doi:10.3389/fonc.2020.590755)
Supplement: Supplementary file 7 [file DataSheet_7.docx]

Supplementary material

S1 Table. Pathological stage, mutation status and smoking status for 186 adenocarcinoma samples provided with gene expression data

S2 Table. List of proteins included and the types of antibodies used in the RPPA analysis.

S3 Table. List of antibodies used for IHC

S4 Table. Proteins significantly differentially expressed (FDR<0.05) in samples from subcluster 2a compared to the other subclusters

S5 Table. Proteins significantly associated with RFS in the total group, in subgroup 2a, and after stratification for smoking- and mutational status

S6 Table. Staining of neuroendocrine markers in samples from subcluster 2a

S7 Table. Proteins associated with RFS were examined using bootstrapping method

S8 Table. Spearman Rank correlation between gene expression and expression of proteins/phosphoproteins

Table S1. Pathological stage, mutation status and smoking status for 186 samples provided with gene expression data.

| **Condition** | | **Number** |
| --- | --- | --- |
| *EGFR* | *Mutated (WT) (nd)* | 24 (159) (3) |
|  |  |  |
| *KRAS* | *Mutated (WT)(nd)* | 67 (109) (10) |
|  |  |  |
| *TP53* | *Mutated (WT) (nd)* | 75 (110) (1) |
|  |  |  |
| Smoking history | *Never (current or former)(nd)* | 18 (166) (2) |
|  |  |  |
| Stage | *Ia/Ib* | 110 |
|  | *IIa/IIB* | 40 |
|  | *IIIa* | 33 |
|  | *nd* | 3 |

Table S2. List of proteins included and the types of antibodies used in the RPPA analysis.

Antibody status for RPPA

(V) = Validated antibody for RPPA

(C) = Use with caution. Validation in progress

(Q) = These antibodies recognize unidentified "damaged" component(s) in addition to its specific protein. The "damaged" component(s) were observed only in certain tissue samples.

(E) = Under Evaluation

(M) = Mouse antibody was used

(G) = Goat antibody was used

(R) = Rabbit antibody was used

(T) = Rat antibody was used

| Protein | Gene | Supplier | Cat # | Species |
| --- | --- | --- | --- | --- |
| 14-3-3-beta | YWHAB | Santa Cruz | sc-628 | Rabbit |
| 14-3-3-epsilon | YWHAE | Santa Cruz | sc-23957 | Mouse |
| 14-3-3-zeta | YWHAZ | Santa Cruz | sc-1019 | Rabbit |
| 4E-BP1 | EIF4EBP1 | CST | 9452 | Rabbit |
| 4E-BP1_pS65 | EIF4EBP1 | CST | 9456 | Rabbit |
| 4E-BP1_pT37_T46 | EIF4EBP1 | CST | 9459 | Rabbit |
| 53BP1 | TP53BP1 | CST | 4937 | Rabbit |
| ACC_pS79 | ACACA | CST | 3661 | Rabbit |
| ACC1 | ACACA | Abcam | ab45174 | Rabbit |
| ACVRL1 | ACVRL1 | Abcam | ab108207 | Rabbit |
| ADAR1 | ADAR | Abcam | ab88574 | Mouse |
| Akt | AKT1 | CST | 4691 | Rabbit |
| Akt_pS473 | AKT1 | CST | 9271 | Rabbit |
| Akt_pT308 | AKT1 | CST | 2965 | Rabbit |
| AMPKa | PRKAA1 | CST | 2532 | Rabbit |
| AMPKa_pT172 | PRKAA1 | CST | 2535 | Rabbit |
| Annexin-I | ANXA1 | BD Biosciences | 610066 | Mouse |
| Annexin-VII | ANXA7 | BD Biosciences | 610668 | Mouse |
| AR | AR | Abcam | ab52615 | Rabbit |
| A-Raf | ARAF | CST | 4432 | Rabbit |
| ARHI | DIRAS3 | MDACC Laboratory | Bast Lab | Mouse |
| ARID1A | ARID1A | Sigma-Aldrich | HPA005456 | Rabbit |
| Atg3 | ATG3 | CST | 3415 | Rabbit |
| Atg7 | ATG7 | CST | 8558 | Rabbit |
| ATM | ATM | CST | 2873 | Rabbit |
| ATM_pS1981 | ATM | CST | 5883 | Rabbit |
| ATP5A | ATP5A | Abcam | ab14748 | Mouse |
| ATR_pS428 | ATR | Abcam | ab178407 | Rabbit |
| Aurora-B | AIM1 | CST | 3094 | Rabbit |
| Axl | AXL | CST | 8661 | Rabbit |
| B7-H3 | CD276 | CST | 14058 | Rabbit |
| B7-H4 | VTCN1 | CST | 14572 | Rabbit |
| b-Actin | ACTB | CST | 4970 | Rabbit |
| Bad_pS112 | BAD | CST | 9291 | Rabbit |
| Bak | BAK1 | Abcam | ab32371 | Rabbit |
| BAP1 | BAP1 | Santa Cruz | sc-28383 | Mouse |
| Bax | BAX | CST | 2772 | Rabbit |
| b-Catenin | CTNNB1 | CST | 9562 | Rabbit |
| b-Catenin_pT41_S45 | CTNNB1 | CST | 9565 | Rabbit |
| Bcl2 | BCL2 | Dako | M0887 | Mouse |
| Bcl2A1 | BCL2A1 | Abnova | PAB8528 | Rabbit |
| Bcl-xL | BCL2L1 | CST | 2762 | Rabbit |
| Beclin | BECN1 | Santa Cruz | sc-10086 | Goat |
| Bid | BID | Abcam | ab32060 | Rabbit |
| Bim | BCL2L11 | Abcam | ab32158 | Rabbit |
| B-Raf | BRAF | Abcam | ab33899 | Rabbit |
| B-Raf_pS445 | BRAF | CST | 2696 | Rabbit |
| BRD4 | BRD4 | CST | 13440 | Rabbit |
| c-Abl | ABL1 | CST | 2862 | Rabbit |
| Caspase-3 | CASP3 | Abcam | ab32042 | Rabbit |
| Caspase-7-cleaved | CASP7 | CST | 9491 | Rabbit |
| Caveolin-1 | CAV1 | CST | 3238 | Rabbit |
| CD171 | L1CAM | BioLegend | 826701 | Mouse |
| CD26 | DPP4 | Abcam | ab28340 | Rabbit |
| CD29 | CD29 | BD Biosciences | 610467 | Mouse |
| CD31 | PECAM1 | Dako | M0823 | Mouse |
| CD44 | CD44 | CST | 3570 | Mouse |
| CD49b | ITGA2 | BD Biosciences | 611016 | Mouse |
| cdc25C | CDC25C | CST | 4688 | Rabbit |
| CDK1 | CDK1 | Abcam | ab32384 | Rabbit |
| Chk1 | CHEK1 | CST | 2360 | Mouse |
| Chk1_pS296 | CHEK1 | Abcam | ab79758 | Rabbit |
| Chk1_pS345 | CHEK1 | CST | 2348 | Rabbit |
| Chk2 | CHEK2 | CST | 3440 | Mouse |
| Chk2_pT68 | CHEK2 | CST | 2197 | Rabbit |
| c-Jun_pS73 | JUN | CST | 9164 | Rabbit |
| c-Kit | KIT | Abcam | ab32363 | Rabbit |
| Claudin-7 | CLDN7 | Novus Biologicals | NB100-91714 | Rabbit |
| c-Met_pY1234_Y1235 | MET | CST | 3129 | Rabbit |
| c-Myc | MYC | Santa Cruz | sc-764 | Rabbit |
| COG3 | COG3 | ProteinTech | 11130-1-AP | Rabbit |
| Collagen-VI | COL6A1 | Santa Cruz | sc-20649 | Rabbit |
| Complex-II-Subunit | SDHB | Invitrogen | 459230 | Mouse |
| Connexin-43 | CNST43 | CST | 3512 | Rabbit |
| Coup-TFII | NR2F2 | CST | 6434 | Rabbit |
| Cox2 | CMC2 | CST | 4842 | Rabbit |
| Cox-IV | PTGS3 | Abcam | ab14744 | Mouse |
| C-Raf | RAF1 | Millipore | 04-739 | Rabbit |
| C-Raf_pS338 | RAF1 | CST | 9427 | Rabbit |
| CXCR4 | CXCR4 | Abcam | ab2074 | Rabbit |
| Cyclin-B1 | CCNB1 | Epitomics | 1495-1 | Rabbit |
| Cyclin-D1 | CCND1 | Santa Cruz | sc-718 | Rabbit |
| Cyclin-D3 | CCND3 | CST | 2936 | Mouse |
| Cyclin-E1 | CCNE1 | Santa Cruz | sc-247 | Mouse |
| Cyclophilin-F | PPIF | Abcam | ab110324 | Mouse |
| Cytokeratin-19 | KRT19 | Dako | M0888 | Mouse |
| D-a-Tubulin | TUBA1A | Abcam | ab48389 | Rabbit |
| DJ1 | PARK7 | Abcam | ab76008 | Rabbit |
| DM-Histone-H3 | HISTH3 | Millipore | 07-030 | Rabbit |
| DM-K9-Histone-H3 | H3K9ME2 | Abcam | ab32521 | Rabbit |
| DUSP4 | DUSP4 | CST | 5149 | Rabbit |
| Dvl3 | DVL3 | CST | 3218 | Rabbit |
| E2F1 | E2F1 | Santa Cruz | sc-251 | Mouse |
| E-Cadherin | CDH1 | CST | 3195 | Rabbit |
| eEF2 | EEF2 | CST | 2332 | Rabbit |
| eEF2K | EEF2K | CST | 3692 | Rabbit |
| EGFR | EGFR | CST | 2232 | Rabbit |
| EGFR_pY1068 | EGFR | CST | 2234 | Rabbit |
| EGFR_pY1173 | EGFR | Abcam | ab32578 | Rabbit |
| eIF4A1 | EIF4A1 | # | # | # |
| eIF4E | EIF4E | CST | 9742 | Rabbit |
| eIF4E_pS209 | EIF4E | Abcam | ab76256 | Rabbit |
| eIF4G | EIF4G1 | CST | 2498 | Rabbit |
| Elk1_pS383 | ELK1 | CST | 9181 | Rabbit |
| EMA | EMA | Dako | M061329-2 | Mouse |
| ER | ESR1 | Lab Vision | RM-9101 | Rabbit |
| ERCC1 | ERCC1 | Santa Cruz | sc-17809 | Mouse |
| ERCC5 | ERCC5 | ProteinTech | 11331-1-AP | Rabbit |
| Ets-1 | ETS1 | Bethyl | A303-501A | Rabbit |
| FAK | PTK2 | Abcam | ab40794 | Rabbit |
| FAK_pY397 | PTK2 | CST | 3283 | Rabbit |
| FASN | FASN | CST | 3180 | Rabbit |
| Fibronectin | FN1 | Epitomics | 1574-1 | Rabbit |
| FoxM1 | FOXM1 | CST | 5436 | Rabbit |
| FoxO3a | FOX3 | CST | 2497 | Rabbit |
| FoxO3a_pS318_S321 | FOXO3 | CST | 9465 | Rabbit |
| FRA-1 | FOSL1 | Santa Cruz | sc-605 | Rabbit |
| G6PD | G6PD | Santa Cruz | sc-373887 | Mouse |
| Gab2 | GAB2 | CST | 3239 | Rabbit |
| GAPDH | GAPDH | Life Technologies | AM4300 | Mouse |
| GATA3 | GATA3 | BD Biosciences | 558686 | Mouse |
| GCN5L2 | KAT2A | CST | 3305 | Rabbit |
| Glutamate-D1-2 | GLUD | CST | 12793 | Rabbit |
| Glutaminase | GLS | Abcam | ab156876 | Rabbit |
| GPBB | PYGM | Novus Biologicals | NBP1-32799 | Rabbit |
| GSK-3a-b | GSK3A/GSK3B | Santa Cruz | sc-7291 | Mouse |
| GSK-3a-b_pS21_S9 | GSK3A GSK3B | CST | 9331 | Rabbit |
| Gys | GYS1 | CST | 3886 | Rabbit |
| Gys_pS641 | GYS1 | CST | 3891 | Rabbit |
| H2AX_pS140 | H2AFX | Pierce Biotechnology | MA1-2022 | Mouse |
| HER2 | ERBB2 | Lab Vision | MS-325-P1 | Mouse |
| HER2_pY1248 | ERBB2 | R&D Systems | AF1768 | Rabbit |
| HER3 | ERBB3 | Santa Cruz | sc-285 | Rabbit |
| HER3_pY1289 | ERBB3 | CST | 4791 | Rabbit |
| Heregulin | NRG1 | CST | 2573 | Rabbit |
| HES1 | HES1 | CST | 11988 | Rabbit |
| Hexokinase-II | HK2 | CST | 2867 | Rabbit |
| HIAP | BIRC2 | Millipore | 07-759 | Rabbit |
| Hif-1-alpha | HIF1A | BD Biosciences | 610958 | Mouse |
| Histone-H3 | HIST3H3 | Abcam | ab1791 | Rabbit |
| HSP27 | HSBP1 | CST | 2402 | Mouse |
| HSP27_pS82 | HSBP1 | CST | 2401 | Rabbit |
| HSP70 | HSPA1A | CST | 4872 | Rabbit |
| IGF1R_pY1135_Y1136 | IGF1R | CST | 3024 | Rabbit |
| IGFBP2 | IGFBP2 | CST | 3922 | Rabbit |
| IGFBP5 | IGFBP5 | Santa Cruz | sc-6006 | Goat |
| IGFRb | IGF1R | CST | 3027 | Rabbit |
| INPP4b | INPP4B | CST | 4039 | Rabbit |
| IRF-1 | IRF1 | Santa Cruz | sc-497 | Rabbit |
| IRS1 | IRS1 | Millipore | 06-248 | Rabbit |
| JAB1 | JAB1 | Santa Cruz | sc-13157 | Mouse |
| Jagged1 | JAG1 | Abcam | ab109536 | Rabbit |
| Jak2 | JAK2 | CST | 3230 | Rabbit |
| JNK_pT183_Y185 | MAPK8 | CST | 4668 | Rabbit |
| JNK2 | MAPK9 | CST | 4672 | Rabbit |
| LC3A-B | LC3AB | CST | 4108 | Rabbit |
| Lck | LCK | CST | 2752 | Rabbit |
| LDHA | LDHA | CST | 3582 | Rabbit |
| MAPK_pT202_Y204 | MAPK3 | CST | 4377 | Rabbit |
| Mcl-1 | MCL1 | CST | 5453 | Rabbit |
| MCT4 | SLC16A4 | Millipore | AB3314P | Rabbit |
| MDM2_pS166 | MDM2 | CST | 3521 | Rabbit |
| MEK1 | MAP2K1 | Epitomics | 1235-1 | Rabbit |
| MEK1_pS217_S221 | MAP2K1 | CST | 9154 | Rabbit |
| MEK2 | MAP2K2 | CST | 9125 | Rabbit |
| Merlin | NF2 | Novus Biologicals | 22710002 | Rabbit |
| MIF | MIF | Santa Cruz | sc-20121 | Rabbit |
| MIG6 | ERRFI1 | Sigma-Aldrich | WH0054206M1 | Mouse |
| Mitochondria | MTCO2 | Abcam | ab3298 | Mouse |
| MMP2 | MMP2 | CST | 4022 | Rabbit |
| Mnk1 | MKNK1 | CST | 2195 | Rabbit |
| MSH2 | MSH2 | CST | 2850 | Mouse |
| MSH6 | MSH6 | Novus Biologicals | 22030002 | Rabbit |
| mTOR | MTOR | CST | 2983 | Rabbit |
| mTOR_pS2448 | MTOR | CST | 2971 | Rabbit |
| Myosin-11 | MYH11 | Novus Biologicals | 21370002 | Rabbit |
| Myosin-IIa_pS1943 | MYO2A | CST | 5026 | Rabbit |
| Myt1 | MYT1 | CST | 4282 | Rabbit |
| NAPSIN-A | NAPSA | Abcam | ab129189 | Rabbit |
| N-Cadherin | CDH2 | CST | 4061 | Rabbit |
| NDRG1_pT346 | NDRG1 | CST | 3217 | Rabbit |
| NDUFB4 | NDUFB4 | Abcam | ab110243 | Mouse |
| NF-kB-p65_pS536 | NFKB1 | CST | 3033 | Rabbit |
| Notch1 | NOTCH1 | CST | 3268 | Rabbit |
| Notch3 | NOTCH3 | Santa Cruz | sc-5593 | Rabbit |
| N-Ras | NRAS | Santa Cruz | sc-31 | Mouse |
| p16INK4a | CDKN2A | Abcam | ab81278 | Rabbit |
| p21 | CDKN1A | Santa Cruz | sc-397 | Rabbit |
| p27_pT157 | CDKN1B | R&D Systems | AF1555 | Rabbit |
| p27_pT198 | CDKN1B | Abcam | ab64949 | Rabbit |
| p27-Kip-1 | CDKN1B | Abcam | ab32034 | Rabbit |
| p38_pT180_Y182 | MAPK14 | CST | 9211 | Rabbit |
| p38-MAPK | MAPK14 | CST | 9212 | Rabbit |
| p44-42-MAPK | MAPK3 | CST | 4695 | Rabbit |
| p53 | TP53 | CST | 9282 | Rabbit |
| p70-S6K_pT389 | RPS6KB1 | CST | 9205 | Rabbit |
| p70-S6K1 | RPS6KB1 | Abcam | ab32529 | Rabbit |
| p90RSK_pT573 | RPS6K | CST | 9346 | Rabbit |
| PAI-1 | SERPINE1 | BD Biosciences | 612024 | Mouse |
| PAR | PAR | Trevigen | 4336-BPC-100 | Rabbit |
| PARP1 | PARP1 | Santa Cruz | sc-7150 | Rabbit |
| Paxillin | PXN | Epitomics | 1500-1 | Rabbit |
| P-Cadherin | CDH3 | CST | 2130 | Rabbit |
| PCNA | PCNA | Abcam | ab29 | Mouse |
| Pdcd-1L1 | PDCD1 | Santa Cruz | sc-19090 | Goat |
| Pdcd4 | PDCD4 | Rockland | 600-401-965 | Rabbit |
| PDGFR-b | PDGFR | CST | 3169 | Rabbit |
| PDK1 | PDK1 | CST | 3062 | Rabbit |
| PDK1_pS241 | PDK1 | CST | 3061 | Rabbit |
| PD-L1 | CD274 | CST | 13684 | Rabbit |
| PEA-15 | PEA15 | CST | 2780 | Rabbit |
| PEA-15_pS116 | PEA15 | Invitrogen | 44-836G | Rabbit |
| PI3K-p110-a | PIK3C2A | CST | 4255 | Rabbit |
| PI3K-p110-b | PIK3BC | Santa Cruz | sc-376412 | Mouse |
| PI3K-p85 | PIK3R1 | Millipore | 06-195 | Rabbit |
| PKA-a | PRKAR1A | CST | 5675 | Rabbit |
| PKC-a | PRKCA | Millipore | 05-154 | Mouse |
| PKC-a_pS657 | PRKCA | Millipore | 06-822 | Rabbit |
| PKC-b-II_pS660 | PRKCB | CST | 9371 | Rabbit |
| PKC-delta_pS664 | PRKCD | Millipore | 07-875 | Rabbit |
| PKM2 | PKM2 | CST | 4053 | Rabbit |
| PLC-gamma2_pY759 | PLCG2 | CST | 3874 | Rabbit |
| PLK1 | PLK1 | CST | 4513 | Rabbit |
| PMS2 | PMS2 | Novus Biologicals | 22510002 | Rabbit |
| Porin | VDAC1 | Abcam | ab14734 | Mouse |
| PR | PGR | Abcam | ab32085 | Rabbit |
| PRAS40 | AKT1S1 | Invitrogen | AHO1031 | Mouse |
| PRAS40_pT246 | AKT1S1 | Life Technologies | 441100G | Rabbit |
| PREX1 | PREX1 | Abcam | ab102739 | Rabbit |
| PTEN | PTEN | CST | 9552 | Rabbit |
| Puma | BBC3 | CST | 4976 | Rabbit |
| PYGM | PYGM | Novus Biologicals | H00005837-M10 | Mouse |
| Rab11 | RAB11A | CST | 3539 | Rabbit |
| Rab25 | RAB25 | CST | 4314 | Rabbit |
| Rad50 | RAD50 | Millipore | 05-525 | Mouse |
| Rad51 | RAD51 | CST | 8875 | Rabbit |
| Raptor | RPTOR | CST | 2280 | Rabbit |
| Rb_pS807_S811 | RB1 | CST | 9308 | Rabbit |
| RBM15 | RBM15 | Novus Biologicals | 21390002 | Rabbit |
| Rheb | RHEB | R&D Systems | MAB3426 | Mouse |
| Rictor | RICTOR | CST | 2114 | Rabbit |
| Rictor_pT1135 | RICTOR | CST | 3806 | Rabbit |
| Rock-1 | ROCK1 | Santa Cruz | sc-5560 | Rabbit |
| RPA32 | RPA32 | CST | 2208 | Rat |
| RPA32_pS4_S8 | RPA32 | Bethyl | A300-245A | Rabbit |
| RSK | RPS6KA1 | CST | 9347 | Rabbit |
| S6 | RPS6 | CST | 2317 | Mouse |
| S6_pS235_S236 | RPS6 | CST | 2211 | Rabbit |
| S6_pS240_S244 | RPS6 | CST | 2215 | Rabbit |
| SCD | SCD | Santa Cruz | sc-58420 | Mouse |
| SDHA | SDHA | CST | 11998 | Rabbit |
| SF2 | SRSF1 | Invitrogen | 32-4500 | Mouse |
| Shc_pY317 | SHC1 | CST | 2431 | Rabbit |
| SHP-2_pY542 | PTPN11 | CST | 3751 | Rabbit |
| SLC1A5 | SLC1A5 | Sigma-Aldrich | HPA035240 | Rabbit |
| Smad1 | SMAD1 | Abcam | ab33902 | Rabbit |
| Smad3 | SMAD3 | Abcam | ab40854 | Rabbit |
| Smad4 | SMAD4 | Santa Cruz | sc-7966 | Mouse |
| Sox2 | SOX2 | CST | 2748 | Rabbit |
| Src | SRC | Millipore | 05-184 | Mouse |
| Src_pY416 | SRC | CST | 2101 | Rabbit |
| Src_pY527 | SRC | CST | 2105 | Rabbit |
| Stat3 | STAT3 | CST | 4904 | Rabbit |
| Stat3_pY705 | STAT3 | CST | 9131 | Rabbit |
| Stat5a | STAT5A | Abcam | ab32043 | Rabbit |
| Stathmin-1 | STMN1 | Abcam | ab52630 | Rabbit |
| Syk | SYK | Santa Cruz | sc-1240 | Mouse |
| Tau | MAPT | Millipore | 05-348 | Mouse |
| TAZ | TAZ | CST | 4883 | Rabbit |
| TFAM | TFAM | CST | 7495 | Rabbit |
| TFRC | TFRC | Novus Biologicals | 22500002 | Rabbit |
| TIGAR | TIGAR | Abcam | ab137573 | Rabbit |
| Transglutaminase | TGM2 | Lab Vision | MS-224-P1 | Mouse |
| TSC1 | TSC1 | CST | 4906 | Rabbit |
| TTF1 | TTF1 | Abcam | ab76013 | Rabbit |
| Tuberin | TSC2 | Abcam | ab32554 | Rabbit |
| Tuberin_pT1462 | TSC2 | CST | 3617 | Rabbit |
| TWIST | TWIST1 | Santa Cruz | sc-81417 | Mouse |
| Tyro3 | TYRO3 | CST | 5585 | Rabbit |
| UBAC1 | UBAC1 | Sigma-Aldrich | HPA005651 | Rabbit |
| Ubq-Histone-H2B | H2BFM | Millipore | 05-1312 | Mouse |
| UGT1A | UGT1A | Santa Cruz | sc-271268 | Mouse |
| VEGFR-2 | KDR | CST | 2479 | Rabbit |
| VHL | VHL | BD Biosciences | 556347 | Mouse |
| Vimentin | VIM | Dako | M0725 | Mouse |
| Wee1 | WEE1 | CST | 4936 | Rabbit |
| XBP-1 | XBP1 | Santa Cruz | sc-32136 | Goat |
| XIAP | XIAP | CST | 2042 | Rabbit |
| XPA | XPA | Santa Cruz | sc-56813 | Mouse |
| XPF | XPF | Abcam | ab3299 | Mouse |
| XRCC1 | XRCC1 | CST | 2735 | Rabbit |
| YAP | YAP1 | Santa Cruz | sc-15407 | Rabbit |
| YAP_pS127 | YAP1 | CST | 4911 | Rabbit |
| YB1 | YBX1 | Novus Biologicals | 17250002 | Rabbit |
| YB1_pS102 | YBX1 | CST | 2900 | Rabbit |

Table S3 Antibodies used for IHC analyses of CD56, synaptophysin, chromograninA and NSE. Catalog number and supplier are included.

| **Marker** | **Antibody** | **Cat.nr** | **Supplier** |
| --- | --- | --- | --- |
| **CD56** | Monoclonal rabbit (clone MRQ-42) | NCL-CD56-1B6 | Leica |
| **Synaptophysin** | Monoclonal mouse (clone DAK-SYNAP) | A0010, Lot.4746 | Dako |
| **Chromogranin A** | Monoclonal mouse (clone LK2H10(9)) | 1199021 | Boehringer Mannheim |
| **NSE** | Monoclonal mouse (clone BBS/NC/VI-H14) | M0873, Lot. 31151 | Dako |

Table S4 Proteins significantly differentially expressed (FDR<0.05) in samples from subcluster 2a compared to the other subclusters(Wilcoxen Rank test).

| **Protein** | **p-value** | **Adjusted p-value** | **Protein** | **p-value** | **Adjusted p-value** |
| --- | --- | --- | --- | --- | --- |
| **Stat5a** | 4.57E-07 | 9.92E-05 | **PKM2** | 0.001 | 0.006 |
| **Rictor** | 1.01E-06 | 9.92E-05 | **Mnk1** | 0.001 | 0.007 |
| **Tuberin** | 1.01E-06 | 9.92E-05 | **Dvl3** | 0.002 | 0.008 |
| **b.Catenin** | 1.65E-06 | < 0.001 | **B.Raf** | 0.002 | 0.008 |
| **PKC.b.II_pS660** | 1.77E-06 | < 0.001 | **Heregulin** | 0.002 | 0.008 |
| **MEK2** | 2.50E-06 | < 0.001 | **Histone.H3** | 0.002 | 0.008 |
| **S6_pS240_S244** | 3.07E-06 | < 0.001 | **LC3A.B** | 0.002 | 0.008 |
| **Jak2** | 4.91E-06 | < 0.001 | **MDM2_pS166** | 0.002 | 0.008 |
| **N.Ras** | 8.32E-06 | < 0.001 | **ATM** | 0.002 | 0.008 |
| **COG3** | 1.08E-05 | < 0.001 | **p90RSK_pT573** | 0.002 | 0.008 |
| **Gab2** | 1.08E-05 | < 0.001 | **Glutamate.D1.2** | 0.002 | 0.009 |
| **Vimentin** | 1.08E-05 | < 0.001 | **ARID1A** | 0.002 | 0.009 |
| **UGT1A** | 1.15E-05 | < 0.001 | **JNK_pT183_Y185** | 0.002 | 0.009 |
| **Axl** | 1.23E-05 | < 0.001 | **XPF** | 0.002 | 0.009 |
| **Cyclophilin.F** | 1.23E-05 | < 0.001 | **Rab11** | 0.003 | 0.010 |
| **Akt_pS473** | 1.91E-05 | < 0.001 | **Cox2** | 0.003 | 0.011 |
| **PKC.a_pS657** | 2.16E-05 | < 0.001 | **PTEN** | 0.003 | 0.011 |
| **Cyclin.E1** | 2.45E-05 | < 0.001 | **Tuberin_pT1462** | 0.003 | 0.012 |
| **eEF2K** | 2.61E-05 | < 0.001 | **Bax** | 0.004 | 0.012 |
| **HER2** | 2.95E-05 | < 0.001 | **Bcl2A1** | 0.004 | 0.012 |
| **Cox.IV** | 3.33E-05 | < 0.001 | **Myosin.11** | 0.004 | 0.012 |
| **Tau** | 3.77E-05 | 0.001 | **TWIST** | 0.004 | 0.012 |
| **B7.H4** | 4.00E-05 | 0.001 | **Smad4** | 0.004 | 0.013 |
| **ATR_pS428** | 4.79E-05 | 0.001 | **Bad_pS112** | 0.004 | 0.014 |
| **mTOR** | 5.09E-05 | 0.001 | **JAB1** | 0.004 | 0.014 |
| **RSK** | 5.40E-05 | 0.001 | **C.Raf_pS338** | 0.005 | 0.016 |
| **S6_pS235_S236** | 5.40E-05 | 0.001 | **eIF4E** | 0.005 | 0.016 |
| **PKC.delta_pS664** | 6.08E-05 | 0.001 | **p44.42.MAPK** | 0.005 | 0.016 |
| **PKC.a** | 6.45E-05 | 0.001 | **c.Jun_pS73** | 0.005 | 0.016 |
| **Shc_pY317** | 6.45E-05 | 0.001 | **H2AX_pS140** | 0.005 | 0.016 |
| **HER2_pY1248** | 7.26E-05 | 0.001 | **Pdcd4** | 0.005 | 0.016 |
| **Src** | 7.69E-05 | 0.001 | **XRCC1** | 0.005 | 0.016 |
| **Complex.II.Subunit** | 9.16E-05 | 0.001 | **LDHA** | 0.005 | 0.016 |
| **EGFR_pY1068** | < 0.001 | 0.001 | **CD44** | 0.006 | 0.019 |
| **X14.3.3.zeta** | < 0.001 | 0.001 | **Smad3** | 0.006 | 0.019 |
| **FAK_pY397** | < 0.001 | 0.001 | **Ubq.Histone.H2B** | 0.006 | 0.019 |
| **Akt** | < 0.001 | 0.001 | **AR** | 0.007 | 0.020 |
| **MIF** | < 0.001 | 0.001 | **ACVRL1** | 0.007 | 0.020 |
| **X4E.BP1** | < 0.001 | 0.001 | **Cyclin.B1** | 0.007 | 0.020 |
| **Caveolin.1** | < 0.001 | 0.001 | **HER3_pY1289** | 0.007 | 0.021 |
| **Porin** | < 0.001 | 0.002 | **Stathmin.1** | 0.007 | 0.021 |
| **PMS2** | < 0.001 | 0.003 | **Myt1** | 0.008 | 0.023 |
| **RBM15** | < 0.001 | 0.003 | **X14.3.3.beta** | 0.008 | 0.023 |
| **PAI.1** | 0.001 | 0.003 | **MCT4** | 0.008 | 0.023 |
| **SHP.2_pY542** | 0.001 | 0.004 | **PI3K.p85** | 0.009 | 0.025 |
| **PLC.gamma2_pY759** | 0.001 | 0.004 | **E.Cadherin** | 0.009 | 0.026 |
| **Gys** | 0.001 | 0.004 | **N.Cadherin** | 0.010 | 0.026 |
| **Fibronectin** | 0.001 | 0.004 | **mTOR_pS2448** | 0.010 | 0.027 |
| **TSC1** | 0.001 | 0.004 | **PYGM** | 0.010 | 0.027 |
| **Raptor** | 0.001 | 0.004 | **Caspase.3** | 0.011 | 0.029 |
| **NDUFB4** | 0.001 | 0.004 | **G6PD** | 0.013 | 0.035 |
| **PI3K.p110.b** | 0.001 | 0.004 | **BRD4** | 0.014 | 0.035 |
| **C.Raf** | 0.001 | 0.004 | **Chk2_pT68** | 0.014 | 0.035 |
| **JNK2** | 0.001 | 0.004 | **TTF1** | 0.014 | 0.035 |
| **ER** | 0.001 | 0.005 | **CXCR4** | 0.016 | 0.039 |
| **PARP1** | 0.001 | 0.005 | **RPA32_pS4_S8** | 0.016 | 0.039 |
| **IGF1R_pY1135_Y1136** | 0.001 | 0.006 | **FAK** | 0.016 | 0.040 |
| **p70.S6K1** | 0.001 | 0.006 | **Paxillin** | 0.017 | 0.042 |
| **c.Abl** | 0.001 | 0.006 | **CDK1** | 0.019 | 0.046 |
| **Chk1_pS296** | 0.001 | 0.006 | **DJ1** | 0.019 | 0.046 |
| **PDGFR.b** | 0.001 | 0.006 | **c.Met_pY1234_Y1235** | 0.020 | 0.047 |
| **X53BP1** | 0.001 | 0.006 | **YAP** | 0.021 | 0.050 |

Table S5. Proteins significantly associated with RFS in the total group, in subgroup 2a, and after stratification for smoking- and mutational status. Proteins in red are described in the paper.

| **Protein (all samples)** | **Gene** | **rho** | **P-value** | **Protein (smokers)** | **Gene** | **rho** | **P-value** |
| --- | --- | --- | --- | --- | --- | --- | --- |
| **c.Abl** | ABL1 | -0.422 | < 0.001 | **c.Abl** | ABL1 | -0.454 | < 0.001 |
| **PKC.b.II_pS660** | PRKCB | 0.376 | 0.001 | **PKC.b.II_pS660** | PRKCB | 0.435 | < 0.001 |
| **PAI.1** | SERPINE1 | -0.372 | 0.002 | **PKC.a** | PRKCA | 0.432 | < 0.001 |
| **PKC.a** | PRKCA | 0.356 | 0.002 | **MIF** | MIF | -0.416 | 0.001 |
| **PKC.a_pS657** | PRKCA.1 | 0.350 | 0.003 | **PKC.a_pS657** | PRKCA.1 | 0.403 | 0.001 |
| **PKC.delta_pS664** | PRKCD | 0.348 | 0.003 | **PAI.1** | SERPINE1 | -0.393 | 0.002 |
| **MIF** | MIF | -0.346 | 0.003 | **CD26** | DPP4 | 0.386 | 0.002 |
| **LC3A.B** | LC3AB | -0.338 | 0.004 | **PKC.delta_pS664** | PRKCD | 0.377 | 0.002 |
| **Caveolin.1** | CAV1 | 0.323 | 0.006 | **Caveolin.1** | CAV1 | 0.377 | 0.003 |
| **GPBB** | PYGM | 0.323 | 0.006 | **SLC1A5** | SLC1A5 | -0.365 | 0.003 |
| **eEF2K** | EEF2K | 0.322 | 0.007 | **Pdcd.1L1** | PDCD1 | 0.353 | 0.005 |
| **PARP1** | PARP1 | -0.316 | 0.008 | **Jak2** | JAK2 | 0.351 | 0.005 |
| **Fibronectin** | FN1 | -0.315 | 0.008 | **EGFR_pY1068** | EGFR.1 | 0.351 | 0.005 |
| **Jak2** | JAK2 | 0.312 | 0.008 | **GPBB** | PYGM | 0.350 | 0.005 |
| **Vimentin** | VIM | 0.310 | 0.009 | **Vimentin** | VIM | 0.340 | 0.007 |
| **B7.H3** | CD276 | -0.310 | 0.009 | **p44.42.MAPK** | MAPK3.1 | 0.338 | 0.007 |
| **B.Raf** | BRAF | 0.298 | 0.012 | **ER** | ESR1 | 0.328 | 0.009 |
| **Pdcd.1L1** | PDCD1 | 0.298 | 0.012 | **PARP1** | PARP1 | -0.319 | 0.011 |
| **TSC1** | TSC1 | 0.294 | 0.013 | **RSK** | RPS6KA1 | 0.317 | 0.012 |
| **p44.42.MAPK** | MAPK3.1 | 0.293 | 0.014 | **B7.H3** | CD276 | -0.317 | 0.012 |
| **Rictor** | RICTOR | 0.290 | 0.015 | **Rictor** | RICTOR | 0.315 | 0.013 |
| **Lck** | LCK | 0.287 | 0.016 | **Fibronectin** | FN1 | -0.312 | 0.014 |
| **b.Catenin** | CTNNB1 | 0.277 | 0.020 | **HER2_pY1248** | ERBB2.1 | 0.308 | 0.015 |
| **Cyclophilin.F** | PPIF | -0.274 | 0.022 | **PLC.gamma2_pY759** | PLCG2 | -0.305 | 0.016 |
| **RSK** | RPS6KA1 | 0.272 | 0.023 | **GSK.3a.b** | GSK3A.GSK3B | -0.302 | 0.017 |
| **HER2** | ERBB2 | 0.267 | 0.026 | **Stat5a** | STAT5A | 0.301 | 0.017 |
| **EGFR_pY1068** | EGFR.1 | 0.266 | 0.026 | **Gys** | GYS1 | -0.299 | 0.018 |
| **CD26** | DPP4 | 0.266 | 0.026 | **eEF2K** | EEF2K | 0.299 | 0.018 |
| **Stat5a** | STAT5A | 0.265 | 0.026 | **B.Raf** | BRAF | 0.297 | 0.019 |
| **PI3K.p85** | PIK3R1 | 0.264 | 0.027 | **EGFR** | EGFR | 0.296 | 0.020 |
| **COG3** | COG3 | 0.264 | 0.027 | **PI3K.p85** | PIK3R1 | 0.293 | 0.021 |
| **JNK_pT183_Y185** | MAPK8 | -0.263 | 0.028 | **JNK_pT183_Y185** | MAPK8 | -0.285 | 0.025 |
| **SLC1A5** | SLC1A5 | -0.263 | 0.028 | **mTOR** | MTOR | 0.285 | 0.025 |
| **ER** | ESR1 | 0.263 | 0.028 | **PR** | PGR | 0.285 | 0.025 |
| **Axl** | AXL | 0.258 | 0.031 | **Axl** | AXL | 0.281 | 0.027 |
| **Cyclin.B1** | CCNB1 | -0.257 | 0.031 | **TSC1** | TSC1 | 0.280 | 0.027 |
| **XRCC1** | XRCC1 | -0.257 | 0.032 | **b.Catenin** | CTNNB1 | 0.278 | 0.029 |
| **Cyclin.E1** | CCNE1 | -0.254 | 0.034 | **Bim** | BCL2L11 | -0.278 | 0.029 |
| **c.Kit** | KIT | -0.253 | 0.035 | **FoxO3a** | FOX3 | 0.275 | 0.031 |
| **PLC.gamma2_pY759** | PLCG2 | -0.252 | 0.035 | **Cyclin.B1** | CCNB1 | -0.274 | 0.031 |
| **C.Raf** | RAF1 | 0.252 | 0.035 | **PLK1** | PLK1 | -0.273 | 0.032 |
| **RPA32** | RPA32 | 0.251 | 0.036 | **LC3A.B** | LC3AB | -0.271 | 0.033 |
| **HER2_pY1248** | ERBB2.1 | 0.247 | 0.039 | **Rab25** | RAB25 | -0.270 | 0.034 |
| **FAK_pY397** | PTK2.1 | 0.245 | 0.041 | **Lck** | LCK | 0.270 | 0.034 |
| **AR** | AR | 0.241 | 0.044 | **HER2** | ERBB2 | 0.269 | 0.034 |
| **Histone.H3** | HIST3H3 | -0.240 | 0.046 | **Cyclophilin.F** | PPIF | -0.269 | 0.034 |
|  |  |  |  | **AR** | AR | 0.268 | 0.035 |
|  |  |  |  | **PKM2** | PKM2 | -0.264 | 0.038 |
|  |  |  |  | **CDK1** | CDK1 | -0.264 | 0.038 |
|  |  |  |  | **Glutaminase** | GLS | 0.262 | 0.040 |
|  |  |  |  | **b.Actin** | ACTB | 0.262 | 0.040 |
|  |  |  |  | **PDK1_pS241** | PDK1.1 | 0.260 | 0.041 |
|  |  |  |  | **PI3K.p110.a** | PIK3C2A | 0.259 | 0.042 |
|  |  |  |  | **Myosin.11** | MYH11 | 0.257 | 0.044 |
|  |  |  |  | **XBP.1** | XBP1 | 0.256 | 0.044 |
|  |  |  |  | **GATA3** | GATA3 | 0.251 | 0.049 |

| **Protein (never smokers)** | **Gene** | **rho** | **P-value** | **Protein (subcluster 2a)** | **Gene** | **rho** | **P-value** |
| --- | --- | --- | --- | --- | --- | --- | --- |
| **MEK1** | MAP2K1 | 0.854 | 0.007 | **Myosin.11** | MYH11 | -0.753 | 0.019 |
| **Bad_pS112** | BAD | -0.854 | 0.007 | **CD26** | DPP4 | 0.728 | 0.026 |
| **Collagen.VI** | COL6A1 | -0.830 | 0.011 | **Caspase.7.cleaved** | CASP7 | -0.711 | 0.032 |
| **LC3A.B** | LC3AB | -0.805 | 0.016 | **YAP** | YAP1 | -0.703 | 0.035 |
| **CD49b** | ITGA2 | -0.805 | 0.016 | **ER** | ESR1 | -0.686 | 0.041 |
| **VHL** | VHL | 0.805 | 0.016 | **X4E.BP1_pT37_T46** | EIF4EBP1.2 | 0.678 | 0.045 |
| **PRAS40_pT246** | AKT1S1.1 | -0.756 | 0.030 | **YAP_pS127** | YAP1 | -0.577 | 0.104 |
| **p16INK4a** | CDKN2A | -0.756 | 0.030 | **HSP27_pS82** | HSBP1 | -0.552 | 0.123 |
| **TFAM** | TFAM | -0.756 | 0.030 |  |  |  |  |
| **Myosin.11** | MYH11 | -0.732 | 0.039 |  |  |  |  |
| **HIAP** | BIRC2 | 0.732 | 0.039 |  |  |  |  |
| **G6PD** | G6PD | -0.732 | 0.039 |  |  |  |  |
| **PEA.15** | PEA15 | -0.732 | 0.039 |  |  |  |  |
| **COG3** | COG3 | 0.708 | 0.050 |  |  |  |  |
| **YB1_pS102** | YBX1.1 | -0.708 | 0.050 |  |  |  |  |

| **Protein** | **Gene** | **rho** | **P-value** | **Protein** | **Gene** | **rho** | **P-value** |
| --- | --- | --- | --- | --- | --- | --- | --- |
| **(EGFR WT)** |  |  |  | **(EGFR mut)** |  |  |  |
| **c.Abl** | ABL1 | -0.441 | < 0.001 | **Bcl.xL** | BCL2L1 | 0.976 | < 0.001 |
| **PKC.a** | PRKCA | 0.391 | 0.002 | **Stat3** | STAT3 | 0.976 | < 0.001 |
| **PKC.a_pS657** | PRKCA.1 | 0.378 | 0.002 | **Puma** | BBC3 | -0.927 | 0.001 |
| **MIF** | MIF | -0.361 | 0.004 | **p16INK4a** | CDKN2A | -0.903 | 0.002 |
| **PAI.1** | SERPINE1 | -0.359 | 0.004 | **Src** | SRC | -0.878 | 0.004 |
| **SLC1A5** | SLC1A5 | -0.354 | 0.005 | **EMA** | EMA | 0.854 | 0.007 |
| **PKC.b.II_pS660** | PRKCB | 0.353 | 0.005 | **IGFRb** | IGF1R.1 | 0.854 | 0.007 |
| **Vimentin** | VIM | 0.335 | 0.008 | **Gys_pS641** | GYS1.1 | 0.854 | 0.007 |
| **PARP1** | PARP1 | -0.335 | 0.008 | **PKC.delta_pS664** | PRKCD | 0.830 | 0.011 |
| **CD26** | DPP4 | 0.335 | 0.008 | **B7.H3** | CD276 | -0.830 | 0.011 |
| **B.Raf** | BRAF | 0.327 | 0.010 | **MEK2** | MAP2K2 | -0.830 | 0.011 |
| **Pdcd.1L1** | PDCD1 | 0.322 | 0.011 | **N.Ras** | NRAS | -0.830 | 0.011 |
| **eEF2K** | EEF2K | 0.316 | 0.012 | **E2F1** | E2F1 | -0.805 | 0.016 |
| **PKC.delta_pS664** | PRKCD | 0.313 | 0.013 | **CXCR4** | CXCR4 | -0.805 | 0.016 |
| **GPBB** | PYGM | 0.309 | 0.014 | **Cyclophilin.F** | PPIF | -0.781 | 0.022 |
| **EGFR_pY1068** | EGFR.1 | 0.308 | 0.015 | **PYGM** | PYGM.1 | 0.781 | 0.022 |
| **GSK.3a.b** | GSK3A.GSK3B | -0.305 | 0.016 | **Stat3_pY705** | STAT3.1 | 0.781 | 0.022 |
| **HER2_pY1248** | ERBB2.1 | 0.300 | 0.018 | **X4E.BP1_pT37_T46** | EIF4EBP1.2 | 0.781 | 0.022 |
| **p44.42.MAPK** | MAPK3.1 | 0.300 | 0.018 | **PTEN** | PTEN | 0.756 | 0.030 |
| **Caveolin.1** | CAV1 | 0.294 | 0.020 | **FAK_pY397** | PTK2.1 | 0.732 | 0.039 |
| **Jak2** | JAK2 | 0.294 | 0.020 | **JNK2** | MAPK9 | 0.732 | 0.039 |
| **Lck** | LCK | 0.286 | 0.024 | **TTF1** | TTF1 | 0.732 | 0.039 |
| **EGFR** | EGFR | 0.285 | 0.025 | **PMS2** | PMS2 | 0.732 | 0.039 |
| **PR** | PGR | 0.285 | 0.025 | **Chk2_pT68** | CHEK2.1 | -0.732 | 0.039 |
| **LC3A.B** | LC3AB | -0.284 | 0.025 | **CD44** | CD44 | -0.732 | 0.039 |
| **PLC.gamma2_pY759** | PLCG2 | -0.283 | 0.026 | **AR** | AR | 0.708 | 0.050 |
| **NDRG1_pT346** | NDRG1 | -0.279 | 0.028 | **Cox.IV** | PTGS3 | -0.708 | 0.050 |
| **Mnk1** | MKNK1 | 0.277 | 0.029 | **B.Raf_pS445** | BRAF.1 | 0.708 | 0.050 |
| **Rictor** | RICTOR | 0.270 | 0.034 | **c.Met_pY1234_Y1235** | MET | -0.708 | 0.050 |
| **Gys** | GYS1 | -0.266 | 0.037 | **MDM2_pS166** | MDM2 | 0.708 | 0.050 |
| **Stat5a** | STAT5A | 0.265 | 0.038 |  |  |  |  |
| **Fibronectin** | FN1 | -0.261 | 0.040 |  |  |  |  |
| **FoxO3a** | FOX3 | 0.260 | 0.041 |  |  |  |  |
| **C.Raf** | RAF1 | 0.260 | 0.041 |  |  |  |  |
| **ER** | ESR1 | 0.260 | 0.042 |  |  |  |  |
| **JNK_pT183_Y185** | MAPK8 | -0.260 | 0.042 |  |  |  |  |
| **Axl** | AXL | 0.257 | 0.044 |  |  |  |  |
| **Bim** | BCL2L11 | -0.256 | 0.044 |  |  |  |  |
| **Histone.H3** | HIST3H3 | -0.256 | 0.044 |  |  |  |  |
| **PKM2** | PKM2 | -0.255 | 0.046 |  |  |  |  |
| **PI3K.p85** | PIK3R1 | 0.255 | 0.046 |  |  |  |  |
| **Cyclin.B1** | CCNB1 | -0.252 | 0.048 |  |  |  |  |

| **Protein (KRAS WT)** | **Gene** | **rho** | **P-value** |  | **Protein (KRAS mut)** | **Gene** | **rho** | **P-value** |
| --- | --- | --- | --- | --- | --- | --- | --- | --- |
| **PKC.delta_pS664** | PRKCD | 0.567 | < 0.001 |  | **Vimentin** | VIM | 0.541 | 0.002 |
| **B7.H3** | CD276 | -0.519 | < 0.001 |  | **EGFR** | EGFR | 0.461 | 0.009 |
| **Jak2** | JAK2 | 0.447 | 0.003 |  | **PKC.a_pS657** | PRKCA.1 | 0.458 | 0.010 |
| **PKC.b.II_pS660** | PRKCB | 0.444 | 0.003 |  | **CD26** | DPP4 | 0.419 | 0.019 |
| **C.Raf** | RAF1 | 0.407 | 0.007 |  | **PARP1** | PARP1 | -0.415 | 0.020 |
| **ATM** | ATM | 0.386 | 0.011 |  | **c.Abl** | ABL1 | -0.414 | 0.020 |
| **PMS2** | PMS2 | 0.380 | 0.012 |  | **PI3K.p110.a** | PIK3C2A | 0.393 | 0.029 |
| **Fibronectin** | FN1 | -0.380 | 0.012 |  | **p44.42.MAPK** | MAPK3.1 | 0.388 | 0.031 |
| **COG3** | COG3 | 0.378 | 0.012 |  | **PAI.1** | SERPINE1 | -0.380 | 0.035 |
| **c.Abl** | ABL1 | -0.375 | 0.013 |  | **Caveolin.1** | CAV1 | 0.378 | 0.036 |
| **LC3A.B** | LC3AB | -0.375 | 0.013 |  | **PKC.a** | PRKCA | 0.367 | 0.042 |
| **ER** | ESR1 | 0.372 | 0.014 |  | **MIF** | MIF | -0.360 | 0.046 |
| **eEF2K** | EEF2K | 0.370 | 0.015 |  |  |  |  |  |
| **TTF1** | TTF1 | 0.366 | 0.016 |  |  |  |  |  |
| **PI3K.p110.b** | PIK3BC | -0.365 | 0.016 |  |  |  |  |  |
| **Pdcd.1L1** | PDCD1 | 0.364 | 0.016 |  |  |  |  |  |
| **c.Kit** | KIT | -0.361 | 0.017 |  |  |  |  |  |
| **Stathmin.1** | STMN1 | -0.360 | 0.018 |  |  |  |  |  |
| **RPA32** | RPA32 | 0.354 | 0.020 |  |  |  |  |  |
| **Lck** | LCK | 0.350 | 0.022 |  |  |  |  |  |
| **Chk2_pT68** | CHEK2.1 | -0.347 | 0.023 |  |  |  |  |  |
| **PTEN** | PTEN | 0.342 | 0.025 |  |  |  |  |  |
| **HER2** | ERBB2 | 0.335 | 0.028 |  |  |  |  |  |
| **PKC.a** | PRKCA | 0.335 | 0.028 |  |  |  |  |  |
| **ARID1A** | ARID1A | 0.332 | 0.030 |  |  |  |  |  |
| **B.Raf** | BRAF | 0.328 | 0.032 |  |  |  |  |  |
| **UGT1A** | UGT1A | -0.325 | 0.033 |  |  |  |  |  |
| **b.Catenin** | CTNNB1 | 0.322 | 0.035 |  |  |  |  |  |
| **XIAP** | XIAP | 0.319 | 0.037 |  |  |  |  |  |
| **BRD4** | BRD4 | 0.318 | 0.038 |  |  |  |  |  |
| **Cyclin.B1** | CCNB1 | -0.317 | 0.039 |  |  |  |  |  |
| **E.Cadherin** | CDH1 | 0.312 | 0.042 |  |  |  |  |  |
| **GPBB** | PYGM | 0.310 | 0.043 |  |  |  |  |  |
| **Cyclophilin.F** | PPIF | -0.308 | 0.044 |  |  |  |  |  |
| **JNK_pT183_Y185** | MAPK8 | -0.304 | 0.047 |  |  |  |  |  |
| **PAI.1** | SERPINE1 | -0.303 | 0.048 |  |  |  |  |  |
| **ERCC5** | ERCC5 | 0.303 | 0.049 |  |  |  |  |  |
| **A.Raf** | ARAF | 0.302 | 0.049 |  |  |  |  |  |

| **Protein (p53 WT)** | **Gene** | **rho** | **P-value** | **Protein (p53 mut)** | **Gene** | **rho** | **P-value** |
| --- | --- | --- | --- | --- | --- | --- | --- |
| **p44.42.MAPK** | MAPK3.1 | 0.501 | 0.001 | **c.Abl** | ABL1 | -0.484 | 0.007 |
| **PKC.b.II_pS660** | PRKCB | 0.486 | 0.001 | **EMA** | EMA | -0.472 | 0.009 |
| **GPBB** | PYGM | 0.485 | 0.002 | **Histone.H3** | HIST3H3 | -0.457 | 0.011 |
| **PAI.1** | SERPINE1 | -0.482 | 0.002 | **HER3_pY1289** | ERBB3.1 | -0.456 | 0.011 |
| **b.Catenin** | CTNNB1 | 0.431 | 0.005 | **TSC1** | TSC1 | 0.454 | 0.012 |
| **PKC.delta_pS664** | PRKCD | 0.425 | 0.006 | **FAK** | PTK2 | 0.439 | 0.015 |
| **RSK** | RPS6KA1 | 0.424 | 0.006 | **AMPKa** | PRKAA1 | -0.429 | 0.018 |
| **Fibronectin** | FN1 | -0.424 | 0.006 | **Vimentin** | VIM | 0.411 | 0.024 |
| **B7.H3** | CD276 | -0.411 | 0.008 | **X14.3.3.zeta** | YWHAZ | -0.390 | 0.033 |
| **EGFR_pY1068** | EGFR.1 | 0.406 | 0.009 | **Hexokinase.II** | HK2 | 0.376 | 0.041 |
| **MIF** | MIF | -0.403 | 0.010 | **PCNA** | PCNA | -0.371 | 0.044 |
| **JNK_pT183_Y185** | MAPK8 | -0.398 | 0.011 | **ERCC1** | ERCC1 | -0.369 | 0.045 |
| **Caveolin.1** | CAV1 | 0.390 | 0.013 | **IRS1** | IRS1 | 0.366 | 0.047 |
| **Cyclin.E1** | CCNE1 | -0.389 | 0.013 | **PKC.a_pS657** | PRKCA.1 | 0.364 | 0.048 |
| **NAPSIN.A** | NAPSA | 0.382 | 0.015 | **PKC.a** | PRKCA | 0.363 | 0.049 |
| **c.Abl** | ABL1 | -0.379 | 0.016 |  |  |  |  |
| **p21** | CDKN1A | -0.378 | 0.016 |  |  |  |  |
| **AR** | AR | 0.377 | 0.016 |  |  |  |  |
| **HES1** | HES1 | -0.377 | 0.017 |  |  |  |  |
| **LC3A.B** | LC3AB | -0.364 | 0.021 |  |  |  |  |
| **PKC.a** | PRKCA | 0.363 | 0.021 |  |  |  |  |
| **HER2_pY1248** | ERBB2.1 | 0.360 | 0.023 |  |  |  |  |
| **FoxM1** | FOXM1 | -0.358 | 0.023 |  |  |  |  |
| **Bim** | BCL2L11 | -0.357 | 0.024 |  |  |  |  |
| **Shc_pY317** | SHC1 | 0.355 | 0.025 |  |  |  |  |
| **GATA3** | GATA3 | 0.347 | 0.028 |  |  |  |  |
| **CDK1** | CDK1 | -0.343 | 0.030 |  |  |  |  |
| **PI3K.p110.a** | PIK3C2A | 0.342 | 0.031 |  |  |  |  |
| **Rictor** | RICTOR | 0.341 | 0.031 |  |  |  |  |
| **Jak2** | JAK2 | 0.337 | 0.033 |  |  |  |  |
| **JNK2** | MAPK9 | 0.336 | 0.034 |  |  |  |  |
| **COG3** | COG3 | 0.332 | 0.036 |  |  |  |  |
| **Heregulin** | NRG1 | -0.332 | 0.037 |  |  |  |  |
| **B.Raf** | BRAF | 0.329 | 0.038 |  |  |  |  |
| **Beclin** | BECN1 | 0.329 | 0.038 |  |  |  |  |
| **PKC.a_pS657** | PRKCA.1 | 0.329 | 0.038 |  |  |  |  |
| **eEF2K** | EEF2K | 0.328 | 0.039 |  |  |  |  |
| **Hexokinase.II** | HK2 | -0.327 | 0.039 |  |  |  |  |
| **PARP1** | PARP1 | -0.324 | 0.041 |  |  |  |  |
| **C.Raf** | RAF1 | 0.324 | 0.042 |  |  |  |  |
| **SLC1A5** | SLC1A5 | -0.323 | 0.042 |  |  |  |  |
| **Notch3** | NOTCH3 | -0.323 | 0.042 |  |  |  |  |
| **CD26** | DPP4 | 0.322 | 0.043 |  |  |  |  |
| **Pdcd.1L1** | PDCD1 | 0.316 | 0.047 |  |  |  |  |
| **B.Raf_pS445** | BRAF.1 | 0.315 | 0.048 |  |  |  |  |
| **FAK_pY397** | PTK2.1 | 0.314 | 0.049 |  |  |  |  |
| **Cyclin.B1** | CCNB1 | -0.313 | 0.049 |  |  |  |  |

Table S6. Positiv expression of synaptophysis (SYP) and neuron specific enolase (NSE) for the majority of samples within subcluster 2a. One samples expressed CD56, and one of the sample expressed Chromogranin A (Chrom). Sections with more than 1% positive cells were regarded as positive.

| ID | CD56 | SYP | Chrom | NSE | pStadium | RFS_months | molecular subtype |
| --- | --- | --- | --- | --- | --- | --- | --- |
| 1 | neg | pos (90%) | neg | pos (90%) | IIa | 44 | PI |
| 2 | neg | pos (100%) | neg | pos (90%) | IIa | 14 | PI |
| 3 | neg | neg | neg | neg | IIb | 2 | PP |
| 4 | neg | Pos (70%)weak | neg | pos (50%) | Ib | 16 | PP |
| 5 | neg | pos (60%) | neg | pos (30%) | Ib | 9 | PP |
| 6 | neg | pos (80%) | neg | pos (90%) | IIa | 7 | PI |
| 7 | neg | pos (80%)strong | neg | pos in stroma | IIIa | 5 | TRU |
| 8 | neg | neg | neg | pos (80%) | IIa | 17 | PP |
| 9 | neg | neg | pos (40%) | neg | IIa | 11 | PP |
| 10 | neg | neg | neg | pos (70%) | IIIa | 4 | PP |
| 11 | pos (10%) | pos (40%)strong | neg | pos (20%) | IIIa | 8 | PP |

Table S7. In order to assess the association between the proteins and RFS we used bootstrapping (n=1000 bootstrapping samples) to evaluate the standard deviation of the rho value. The p-value was adjusted using Benjamini-Hochberg procedure.

| Protein | rho | p-value | adjusted p-value | rho.sd | 2.sd | rho>2*SD |
| --- | --- | --- | --- | --- | --- | --- |
| c.Abl | -0.422 | <0.001 | 0.081 | 0.102 | 0.204 | Yes |
| PKC.b.II_pS660 | 0.376 | 0.001 | 0.141 | 0.11 | 0.22 | Yes |
| PAI.1 | -0.372 | 0.002 | 0.141 | 0.107 | 0.214 | Yes |
| PKC.a | 0.356 | 0.002 | 0.141 | 0.107 | 0.214 | Yes |
| MIF | -0.346 | 0.003 | 0.141 | 0.11 | 0.22 | Yes |
| PKC.a_pS657 | 0.35 | 0.003 | 0.141 | 0.108 | 0.216 | Yes |
| PKC.delta_pS664 | 0.348 | 0.003 | 0.141 | 0.119 | 0.238 | Yes |
| LC3A.B | -0.338 | 0.004 | 0.155 | 0.12 | 0.24 | Yes |
| Caveolin.1 | 0.323 | 0.006 | 0.165 | 0.109 | 0.218 | Yes |
| GPBB | 0.323 | 0.006 | 0.165 | 0.121 | 0.242 | Yes |
| eEF2K | 0.322 | 0.007 | 0.165 | 0.11 | 0.22 | Yes |
| Fibronectin | -0.315 | 0.008 | 0.165 | 0.111 | 0.222 | Yes |
| Jak2 | 0.312 | 0.008 | 0.165 | 0.123 | 0.246 | Yes |
| PARP1 | -0.316 | 0.008 | 0.165 | 0.11 | 0.22 | Yes |
| B7.H3 | -0.31 | 0.009 | 0.165 | 0.113 | 0.226 | Yes |
| Vimentin | 0.31 | 0.009 | 0.165 | 0.113 | 0.226 | Yes |
| B.Raf | 0.298 | 0.012 | 0.202 | 0.111 | 0.222 | Yes |
| Pdcd.1L1 | 0.298 | 0.012 | 0.202 | 0.113 | 0.226 | Yes |
| TSC1 | 0.294 | 0.013 | 0.203 | 0.118 | 0.236 | Yes |
| p44.42.MAPK | 0.293 | 0.014 | 0.203 | 0.123 | 0.246 | Yes |
| Rictor | 0.29 | 0.015 | 0.210 | 0.116 | 0.232 | Yes |
| Lck | 0.287 | 0.016 | 0.214 | 0.126 | 0.252 | Yes |
| b.Catenin | 0.277 | 0.02 | 0.243 | 0.118 | 0.236 | Yes |
| Cyclophilin.F | -0.274 | 0.022 | 0.243 | 0.107 | 0.214 | Yes |
| RSK | 0.272 | 0.023 | 0.243 | 0.114 | 0.228 | Yes |
| CD26 | 0.266 | 0.026 | 0.243 | 0.106 | 0.212 | Yes |
| EGFR_pY1068 | 0.266 | 0.026 | 0.243 | 0.112 | 0.224 | Yes |
| HER2 | 0.267 | 0.026 | 0.243 | 0.113 | 0.226 | Yes |
| Stat5a | 0.265 | 0.026 | 0.243 | 0.119 | 0.238 | Yes |
| COG3 | 0.264 | 0.027 | 0.243 | 0.114 | 0.228 | Yes |
| PI3K.p85 | 0.264 | 0.027 | 0.243 | 0.128 | 0.256 | Yes |
| ER | 0.263 | 0.028 | 0.243 | 0.107 | 0.214 | Yes |
| JNK_pT183_Y185 | -0.263 | 0.028 | 0.243 | 0.116 | 0.232 | Yes |
| SLC1A5 | -0.263 | 0.028 | 0.243 | 0.12 | 0.24 | Yes |
| Axl | 0.258 | 0.031 | 0.252 | 0.124 | 0.248 | Yes |
| Cyclin.B1 | -0.257 | 0.031 | 0.252 | 0.106 | 0.212 | Yes |
| XRCC1 | -0.257 | 0.032 | 0.252 | 0.105 | 0.21 | Yes |
| Cyclin.E1 | -0.254 | 0.034 | 0.252 | 0.119 | 0.238 | Yes |
| c.Kit | -0.253 | 0.035 | 0.252 | 0.122 | 0.244 | Yes |
| C.Raf | 0.252 | 0.035 | 0.252 | 0.114 | 0.228 | Yes |
| PLC.gamma2_pY759 | -0.252 | 0.035 | 0.252 | 0.119 | 0.238 | Yes |
| RPA32 | 0.251 | 0.036 | 0.252 | 0.117 | 0.234 | Yes |
| HER2_pY1248 | 0.247 | 0.039 | 0.269 | 0.111 | 0.222 | Yes |
| FAK_pY397 | 0.245 | 0.041 | 0.272 | 0.115 | 0.23 | Yes |
| AR | 0.241 | 0.044 | 0.290 | 0.116 | 0.232 | Yes |
| Histone.H3 | -0.24 | 0.046 | 0.293 | 0.116 | 0.232 | Yes |

Table S8. Spearman Rank correlation was utilized between gene expression and expression of proteins/phosphoproteins. Proteins/genes with the highest correlation are listed first.

| **Gene Name** | **Protein** | **corr.coef.** | **Gene Name** | **Protein** | **corr.coef.** |
| --- | --- | --- | --- | --- | --- |
| **KIT** | c-Kit | 0.815 | **CHEK1** | Chk1_pS296 | 0.239 |
| **IGFBP2** | IGFBP2 | 0.788 | **TYRO3** | Tyro3 | 0.234 |
| **CDK1** | CDK1 | 0.779 | **SCD** | SCD | 0.234 |
| **PRKCA** | PKC-a_pS657 | 0.758 | **UBAC1** | UBAC1 | 0.232 |
| **CCNB1** | Cyclin-B1 | 0.758 | **SRC** | Src | 0.232 |
| **ANXA1** | Annexin-I | 0.754 | **PTK2** | FAK | 0.230 |
| **DUSP4** | DUSP4 | 0.744 | **RHEB** | Rheb | 0.226 |
| **FASN** | FASN | 0.739 | **ETS1** | Ets-1 | 0.226 |
| **EIF4EBP1** | 4E-BP1 | 0.723 | **GATA3** | GATA3 | 0.225 |
| **EGFR** | EGFR | 0.722 | **SMAD3** | Smad3 | 0.225 |
| **DPP4** | CD26 | 0.720 | **ATR** | ATR_pS428 | 0.224 |
| **MSH6** | MSH6 | 0.700 | **PTK2** | FAK_pY397 | 0.222 |
| **GLS** | Glutaminase | 0.699 | **CASP3** | Caspase-3 | 0.220 |
| **CCNE1** | Cyclin-E1 | 0.682 | **ARAF** | A-Raf | 0.217 |
| **CDKN2A** | p16INK4a | 0.678 | **BAP1** | BAP1 | 0.216 |
| **YWHAZ** | 14-3-3-zeta | 0.665 | **SERPINE1** | PAI-1 | 0.216 |
| **PRKCA** | PKC-a | 0.648 | **ERBB2** | HER2_pY1248 | 0.208 |
| **YAP1** | YAP_pS127 | 0.637 | **CHEK2** | Chk2_pT68 | 0.206 |
| **CD274** | PD-L1 | 0.627 | **HSBP1** | HSP27 | 0.206 |
| **RAB25** | Rab25 | 0.623 | **MAP2K2** | MEK2 | 0.205 |
| **LCK** | Lck | 0.615 | **CTNNB1** | b-Catenin | 0.200 |
| **PLK1** | PLK1 | 0.603 | **TSC2** | Tuberin | 0.197 |
| **PDCD4** | Pdcd4 | 0.596 | **MYC** | c-Myc | 0.191 |
| **TFRC** | TFRC | 0.584 | **BBC3** | Puma | 0.190 |
| **GAPDH** | GAPDH | 0.584 | **MAPK9** | JNK2 | 0.187 |
| **HK2** | Hexokinase-II | 0.583 | **PRKCD** | PKC-delta_pS664 | 0.180 |
| **ITGA2** | CD49b | 0.580 | **BCL2A1** | Bcl2A1 | 0.180 |
| **PARP1** | PARP1 | 0.575 | **ERBB2** | HER2 | 0.175 |
| **PKM2** | PKM2 | 0.572 | **FOSL1** | FRA-1 | 0.174 |
| **LDHA** | LDHA | 0.571 | **RAD50** | Rad50 | 0.173 |
| **G6PD** | G6PD | 0.570 | **PECAM1** | CD31 | 0.169 |
| **FN1** | Fibronectin | 0.563 | **ERCC5** | ERCC5 | 0.167 |
| **MYH11** | Myosin-11 | 0.562 | **AKT1S1** | PRAS40 | 0.166 |
| **SOX2** | Sox2 | 0.561 | **XPA** | XPA | 0.164 |
| **CLDN7** | Claudin-7 | 0.561 | **VTCN1** | B7-H4 | 0.164 |
| **ATM** | ATM | 0.557 | **CDH2** | N-Cadherin | 0.156 |
| **MIF** | MIF | 0.549 | **PLCG2** | PLC-gamma2_pY759 | 0.155 |
| **RPS6KA1** | RSK | 0.543 | **PRKCB** | PKC-b-II_pS660 | 0.151 |
| **PREX1** | PREX1 | 0.540 | **BAX** | Bax | 0.151 |
| **NR2F2** | Coup-TFII | 0.528 | **AKT1** | Akt | 0.151 |
| **BCL2** | Bcl2 | 0.523 | **KDR** | VEGFR-2 | 0.151 |
| **SLC1A5** | SLC1A5 | 0.520 | **CASP7** | Caspase-7-cleaved | 0.145 |
| **EIF4E** | eIF4E | 0.518 | **GYS1** | Gys_pS641 | 0.143 |
| **PPIF** | Cyclophilin-F | 0.513 | **TGM2** | Transglutaminase | 0.141 |
| **MSH2** | MSH2 | 0.505 | **L1CAM** | CD171 | 0.137 |
| **YAP1** | YAP | 0.504 | **EGFR** | EGFR_pY1173 | 0.136 |
| **ATG7** | Atg7 | 0.503 | **UGT1A** | UGT1A | 0.135 |
| **AXL** | Axl | 0.502 | **IGF1R** | IGF1R_pY1135_Y1136 | 0.135 |
| **IGF1R** | IGFRb | 0.493 | **MTOR** | mTOR | 0.129 |
| **ACACA** | ACC_pS79 | 0.490 | **ERCC1** | ERCC1 | 0.127 |
| **ACACA** | ACC1 | 0.484 | **TSC2** | Tuberin_pT1462 | 0.122 |
| **HES1** | HES1 | 0.480 | **MDM2** | MDM2_pS166 | 0.118 |
| **AR** | AR | 0.475 | **RICTOR** | Rictor | 0.116 |
| **GYS1** | Gys | 0.474 | **BRD4** | BRD4 | 0.115 |
| **ARID1A** | ARID1A | 0.471 | **ATG3** | Atg3 | 0.112 |
| **CHEK1** | Chk1 | 0.468 | **BIRC2** | HIAP | 0.109 |
| **ERBB3** | HER3 | 0.467 | **NF2** | Merlin | 0.109 |
| **STAT5A** | Stat5a | 0.462 | **EIF4E** | eIF4E_pS209 | 0.102 |
| **CDH1** | E-Cadherin | 0.458 | **HIST3H3** | Histone-H3 | 0.092 |
| **NAPSA** | NAPSIN-A | 0.435 | **KAT2A** | GCN5L2 | 0.090 |
| **XRCC1** | XRCC1 | 0.434 | **PYGM** | PYGM | 0.079 |
| **CDC25C** | cdc25C | 0.428 | **RPTOR** | Raptor | 0.076 |
| **ACTB** | b-Actin | 0.424 | **H2AFX** | H2AX_pS140 | 0.075 |
| **ANXA7** | Annexin-VII | 0.421 | **CHEK1** | Chk1_pS345 | 0.075 |
| **IRS1** | IRS1 | 0.420 | **YWHAE** | 14-3-3-epsilon | 0.074 |
| **NRG1** | Heregulin | 0.418 | **SDHB** | Complex-II-Subunit | 0.064 |
| **EGFR** | EGFR_pY1068 | 0.415 | **PXN** | Paxillin | 0.064 |
| **DVL3** | Dvl3 | 0.415 | **MAPT** | Tau | 0.062 |
| **RPS6** | S6 | 0.404 | **IGFBP5** | IGFBP5 | 0.049 |
| **MCL1** | Mcl-1 | 0.403 | **JAG1** | Jagged1 | 0.047 |
| **SYK** | Syk | 0.401 | **TP53** | p53 | 0.045 |
| **STAT3** | Stat3 | 0.399 | **RBM15** | RBM15 | 0.042 |
| **TP53BP1** | 53BP1 | 0.399 | **BRAF** | B-Raf | 0.041 |
| **JAK2** | Jak2 | 0.398 | **TTF1** | TTF1 | 0.036 |
| **TFAM** | TFAM | 0.390 | **HIF1A** | Hif-1-alpha | 0.032 |
| **INPP4B** | INPP4b | 0.389 | **MAPK8** | JNK_pT183_Y185 | 0.025 |
| **XIAP** | XIAP | 0.388 | **ELK1** | Elk1_pS383 | 0.022 |
| **TSC1** | TSC1 | 0.388 | **DIRAS3** | ARHI | 0.022 |
| **EEF2K** | eEF2K | 0.383 | **BID** | Bid | 0.021 |
| **PIK3R1** | PI3K-p85 | 0.380 | **MYT1** | Myt1 | 0.019 |
| **PRKAA1** | AMPKa | 0.379 | **RICTOR** | Rictor_pT1135 | 0.019 |
| **PDCD1** | Pdcd-1L1 | 0.374 | **YWHAB** | 14-3-3-beta | 0.016 |
| **MAP2K1** | MEK1 | 0.373 | **ATM** | ATM_pS1981 | 0.016 |
| **PCNA** | PCNA | 0.369 | **RAF1** | C-Raf_pS338 | 0.015 |
| **WEE1** | Wee1 | 0.368 | **AKT1** | Akt_pS473 | 0.015 |
| **ADAR** | ADAR1 | 0.365 | **RPS6KB1** | p70-S6K_pT389 | 0.013 |
| **MMP2** | MMP2 | 0.364 | **ROCK1** | Rock-1 | 0.010 |
| **PAR** | PAR | 0.352 | **CTNNB1** | b-Catenin_pT41_S45 | 0.009 |
| **PMS2** | PMS2 | 0.351 | **BCL2L1** | Bcl-xL | 0.007 |
| **COG3** | COG3 | 0.350 | **TWIST1** | TWIST | 0.006 |
| **CDH3** | P-Cadherin | 0.344 | **YBX1** | YB1 | 0.005 |
| **BRAF** | B-Raf_pS445 | 0.344 | **SMAD4** | Smad4 | 0.005 |
| **EIF4EBP1** | 4E-BP1_pT37_T46 | 0.337 | **E2F1** | E2F1 | 0.001 |
| **EIF4G1** | eIF4G | 0.335 | **RAB11A** | Rab11 | 0.000 |
| **RPS6KB1** | p70-S6K1 | 0.328 | **ERRFI1** | MIG6 | 0.000 |
| **CD276** | B7-H3 | 0.325 | **PYGM** | GPBB | -0.002 |
| **VIM** | Vimentin | 0.324 | **AIM1** | Aurora-B | -0.007 |
| **IRF1** | IRF-1 | 0.322 | **RB1** | Rb_pS807_S811 | -0.009 |
| **SMAD1** | Smad1 | 0.321 | **PDK1** | PDK1_pS241 | -0.009 |
| **KRT19** | Cytokeratin-19 | 0.317 | **ERBB3** | HER3_pY1289 | -0.013 |
| **EIF4A1** | eIF4A1 | 0.316 | **FOXM1** | FoxM1 | -0.018 |
| **CHEK2** | Chk2 | 0.312 | **RPS6** | S6_pS235_S236 | -0.026 |
| **SHC1** | Shc_pY317 | 0.309 | **NFKB1** | NF-kB-p65_pS536 | -0.026 |
| **ESR1** | ER | 0.307 | **MET** | c-Met_pY1234_Y1235 | -0.039 |
| **STMN1** | Stathmin-1 | 0.307 | **AKT1** | Akt_pT308 | -0.042 |
| **BCL2L11** | Bim | 0.307 | **MAP2K1** | MEK1_pS217_S221 | -0.048 |
| **GAB2** | Gab2 | 0.304 | **RPS6** | S6_pS240_S244 | -0.051 |
| **PEA15** | PEA-15 | 0.304 | **COL6A1** | Collagen-VI | -0.062 |
| **EIF4EBP1** | 4E-BP1_pS65 | 0.303 | **TAZ** | TAZ | -0.065 |
| **ABL1** | c-Abl | 0.301 | **HSBP1** | HSP27_pS82 | -0.066 |
| **CXCR4** | CXCR4 | 0.299 | **BAD** | Bad_pS112 | -0.066 |
| **PGR** | PR | 0.292 | **HSPA1A** | HSP70 | -0.077 |
| **EEF2** | eEF2 | 0.292 | **YBX1** | YB1_pS102 | -0.078 |
| **H2BFM** | Ubq-Histone-H2B | 0.291 | **RAD51** | Rad51 | -0.079 |
| **CAV1** | Caveolin-1 | 0.286 | **TUBA1A** | D-a-Tubulin | -0.080 |
| **SRC** | Src_pY416 | 0.286 | **PEA15** | PEA-15_pS116 | -0.083 |
| **NOTCH3** | Notch3 | 0.285 | **PRKAA1** | AMPKa_pT172 | -0.087 |
| **RAF1** | C-Raf | 0.282 | **NDUFB4** | NDUFB4 | -0.087 |
| **PTEN** | PTEN | 0.279 | **AKT1S1** | PRAS40_pT246 | -0.089 |
| **MKNK1** | Mnk1 | 0.278 | **PDK1** | PDK1 | -0.095 |
| **NDRG1** | NDRG1_pT346 | 0.276 | **MTOR** | mTOR_pS2448 | -0.103 |
| **CDKN1A** | p21 | 0.272 | **PIK3C2A** | PI3K-p110-a | -0.113 |
| **MAPK14** | p38-MAPK | 0.268 | **MAPK3** | MAPK_pT202_Y204 | -0.116 |
| **STAT3** | Stat3_pY705 | 0.265 | **SRSF1** | SF2 | -0.128 |
| **JUN** | c-Jun_pS73 | 0.263 | **CDKN1B** | p27_pT157 | -0.132 |
| **CCND3** | Cyclin-D3 | 0.263 | **MAPK14** | p38_pT180_Y182 | -0.150 |
| **CDKN1B** | p27-Kip-1 | 0.263 | **PRKAR1A** | PKA-a | -0.158 |
| **NOTCH1** | Notch1 | 0.259 | **BECN1** | Beclin | -0.160 |
| **ACVRL1** | ACVRL1 | 0.254 | **SLC16A4** | MCT4 | -0.183 |
| **FOXO3** | FoxO3a_pS318_S321 | 0.252 | **BAK1** | Bak | -0.190 |
| **SDHA** | SDHA | 0.250 | **CDKN1B** | p27_pT198 | -0.199 |
| **CD29** | CD29 | 0.248 | **XBP1** | XBP-1 | -0.207 |
| **NRAS** | N-Ras | 0.247 | **VHL** | VHL | -0.213 |
| **MAPK3** | p44-42-MAPK | 0.242 | **CCND1** | Cyclin-D1 | -0.253 |
| **SRC** | Src_pY527 | 0.242 | **PTPN11** | SHP-2_pY542 | -0.294 |
